# Supplementary material for: Understanding and applying practitioner and patient views on the implementation of a novel automated Computer-Aided Risk Score (CARS) predicting the risk of death following emergency medical admission to hospital: qualitative study
Source: BMJ Open. 2019 Apr 23;9(4):e026591. doi: 10.1136/bmjopen-2018-026591 (PMC6500336; doi:10.1136/bmjopen-2018-026591)
Supplement: Author's manuscript [file bmjopen-2018-026591.draft_revisions.pdf]

BMJ Open

BMJ Open is committed to open peer review. As part of this commitment we make the peer review history of every article we publish publicly available.

When an article is published we post the peer reviewers' comments and the authors' responses online. We also post the versions of the paper that were used during peer review. These are the versions that the peer review comments apply to.

The versions of the paper that follow are the versions that were submitted during the peer review process. They are not the versions of record or the final published versions. They should not be cited or distributed as the published version of this manuscript.

BMJ Open is an open access journal and the full, final, typeset and author-corrected version of record of the manuscript is available on our site with no access controls, subscription charges or pay-per-view fees (http://bmjopen.bmj.com).

If you have any questions on BMJ Open's open peer review process please email info.bmjopen@bmj.com

BMJ Open

Practitioner and patient involvement in the implementation of a novel automated Computer Aided Risk Score (CARS) predicting the risk of death following emergency medical admission to hospital: A qualitative study

Journal:	*BMJ Open*
Manuscript ID	bmjopen-2018-026591
Article Type:	Research
Date Submitted by the Author:	10-Oct-2018

Complete List of Authors:	Dyson, Judith; University of Hull, Health and Social Work<br>Marsh, Claire; Bradord Institute for Health Research, Quality and Safety<br>Jackson , Natalie ; Bradord Institute for Health Research, Quality and Safety<br>Richardson, Donald; York Teaching Hospital NHS Foundation Trust Hospital, Renal Medicine<br>Faisal, Muhammad; University of Bradford, Faculty of Health Studies; University of Bradford<br>Scally, Andrew ; University of Bradford, School of Health Studies<br>Mohammed, Mohammed ; University of Bradford, School of Health Studies

Keywords:	Computer Aided Risk Score, Implementation, Service User Involvement, CARS, Early warning score, Hospital Mortality

SCHOLARONE™
Manuscripts

Practitioner and patient involvement in the implementation of a novel automated Computer Aided Risk Score (CARS) predicting the risk of death following emergency medical admission to hospital: A qualitative study

Abstract (273 words)

Objectives: To elicit the views of health care practitioners and patients on i) the potential value, unintended consequences and concerns associated with the Computer Aided Risk Score (CARS) and practitioner views on ii) the issues to consider before embedding CARS into routine practice.

Setting: This study was conducted in two NHS Hospital Trusts in the North of England. Both had in-house IT development teams, mature IT infrastructure with electronic National Early Warning Score NEWS) and were capable of integrating NEWS with blood test results. The study focused on emergency medical and elderly admissions units. There were 60 and 39 acute medical/elderly admissions beds at the two NHS hospital Trusts.

Participants: We conducted 8 focus groups with 11 service users and carers (SU/Cs) and 45 health care practitioners in two NHS acute hospitals.

Results: Staff and SU/Cs recognised the potential of CARS but were clear the score should not replace or undermine clinical judgments. Staff recognised CARS could enhance clinical decision making/judgments and aid communication with patients. They wanted to understand the components of CARS and be reassured about its accuracy but were concerned about the impact on intensive care and blood tests.

Conclusion: Risk scores are widely used in healthcare, but their development and implementation does not usually involve input from practitioners and patients. We developed a novel computer aided risk score which estimates the risk of death following emergency admission to medical wards in hospital. CARS relies on routinely collected vital signs and blood test data. Staff and patients have important, often complex, insights to support the development and implementation of CARS are being addressed to ensure CARS is successfully implemented in routine clinical practice.

For peer review only - http://bmjopen.bmj.com/site/about/guidelines.xhtml

Article Summary

- This paper reports the involvement of practice staff and service users/carers in considering the implementation of the Computer Aided Risk Score (CARS) into clinical practice
- Staff recognised such a score could enhance clinical decision making but wanted to understand the components of the score and be reassured about its accuracy
- Service users and carers were cautious such a score might replace clinical judgement or contribute extra workload to hospital staff
- Staff and service users/carers have offered important, complex insights which could support the implementation of risk scores into clinical practice

For peer review only

For peer review only - http://bmjopen.bmj.com/site/about/guidelines.xhtml

Practitioner and patient involvement in the implementation of a novel automated Computer Aided Risk Score (CARS) predicting the risk of death following emergency medical admission to hospital: A qualitative study

Dr Judith Dyson, University of Hull, Cottingham Road, HULL, UK HU6 7RX, T: 01482 463806, E: j.dyson@hull.ac.uk
Dr Claire Marsh, Bradford Institute of Health Research, Bradford UK
Ms Natalie Jackson, Bradford Institute of Health Research, Bradford UK
Dr Donald Richardson, York Teaching Hospitals NHS Foundation Trust, York UK
Dr Muhammad Faisal, University of Bradford, Bradford, UK
R Andrew Scally, University of Bradford, Bradford, UK
Prof Mohammed Mohammed, University of Bradford, Bradford, UK

Key Terms: "Computer Aided Risk Score", CARS, "Service User Involvement", Implementation, "Early Warning Score", "Hospital Mortality"

Word count: 4033

Introduction

Despite the widespread use of risk scores to enhance decision making in healthcare there is little or no evidence of the involvement of healthcare practitioners and/or patients/service users or carers in the design, development and implementation of this type of risk score. For instance, Brabrand et al (2010) identified ten different scoring systems for estimating the risk of in-hospital mortality, but there was little or no mention of the involvement of practitioners and/or patients in the development and implantation of this type of risk scoring systems.

We embarked on the development a novel computer aided risk score (CARS) for estimating the risk of in-hospital mortality following emergency medical admission to hospital (Faisal et al, 2018) in two hospitals. We designed CARS to rely on variables already routinely collected and electronically recorded as part of the process of care including vital signs data (based on a National Early Warning Score (NEWS)) and blood test results (Department of Health 2013). Previously developed risk scores rely on *either* physiological measurements *or* blood test results.

Concurrent to the statistical modelling work, we conducted focus groups with staff and service users and carers (SU/C). Focus groups were designed to feed into the on-going development of CARS. The aims of the focus groups were to establish i) health care practitioner (hereafter "staff") and SU/C views on the potential value, unintended consequences and concerns associated with the development of the CARS and ii) staff views on how CARS should be adopted in practice/implementation needs

For peer review only - http://bmjopen.bmj.com/site/about/guidelines.xhtml

Methods

Ethical approval

Ethical approval was granted from National Research Ethics Service (15/YH/0348) and Research Governance approval from the trusts involved in the study. All participants gave signed consent after receiving written information about the study.

Patient and Public Involvement

The "Service User and Carer Involvement in Research Group" (SURG) at the University of Bradford supported the project as members of the project steering group and as a focus group advisory team. Their contribution included co-design of project materials, support of the methodology (e.g. recruitment strategies) and offering comments and suggestions based on data gathered.

Participants

This study was conducted in two NHS Hospital Trusts in the North of England (referred to hereafter as Trust A and Trust B). Both had in-house IT development teams, mature IT infrastructure with electronic NEWS and were capable of integrating NEWS with blood test results. The study focused on emergency medical and elderly admissions units. There were 60 and 39 acute medical/elderly admissions beds at Trusts A and B respectively.

With respect to SU/Cs were competent adults (aged over 18 years) who were members of the public, who had either been in hospital themselves any time in their adult life, or who had experienced a relative in hospital. Staff were, any practitioner working in areas where we intended to implement CARS (acute assessment units, medical wards and older person in-patient units) or acute outreach staff (nurse or doctor called upon to offer advanced assessment and input before irretrievable deterioration occurs), were eligible. Due to the additional aim of the staff groups (implementation needs) we held separate SU/C and staff focus groups.

Design

Eight focus groups were held over two rounds in each Trust. A first round of staff and SU/C focus groups was conducted at the beginning of CARS development and commenced with a brief presentation about CARS, its rationale and development, then asked participants for their thoughts, feelings and concerns in relation to implementation of a CARS at their hospitals. Focus group schedules were also informed by the literature relating to other risk scores (e.g. NEWS). Results from these focus groups with were fed back to the CARS research team who then further developed CARS and its implementation package (Figure 1 illustrates round one focus group questions). Subsequently, a second round of focus groups were held with staff to explore CARS implementation needs in greater

For peer review only - http://bmjopen.bmj.com/site/about/guidelines.xhtml

depth. After a presentation about the CARS, vignettes were used (developed from case note reviews), to allow staff to understand how CARS scores relate to real clinical scenarios (Figure 2 offers an example of a vignette and illustrates round two focus group questions). It was our intention to run groups of between 6 and 12 participants. Due to the challenges of staff time and availability, this was revised to smaller group sizes for the second staff focus groups. JD led staff focus groups, CM led SU/C groups and NJ supported all groups (none of whom had a previous relationship with participants).

Figure 1: Focus group questions round 1

Figure 2: Focus group content round 2

Procedure

CM approached the patient experience leads at each hospital as a gatekeeper for recruitment to SU/Cs. Patient Experience leads contacted members of their existing forums by email, posters and verbal invites. Interested people contacted CM directly and were then given participant information sheets before deciding to attend. Our clinical partners from the CARS implementation teams at both hospitals introduced JD and NJ (electronically or in person) to the nurse in charge of relevant hospital areas. Charge nurses circulated email invitations to qualified medical and nursing staff of all grades with participant information who were contacted the research team if they were interested in participating.

The first round of focus groups (SU/C and staff) took place over a six-month period from May 2016 and the second staff focus groups occurred between May and July 2017. For staff, purposive sampling identified a diverse range of participants in terms of their professional role and experience. We expected focus groups including 25 participants would be enough to achieve data saturation (Guest et al 2006). With one exception, all focus groups took place in hospital meeting rooms or offices. The exception was one staff focus group, which took place at a conference centre (for the convenience of staff who were attending).

Analysis

Focus groups were audio recorded and transcribed verbatim and transcripts imported into NVivo v.11 data management software. Data were subject to thematic analysis (Braun and Clarke 2006). An inductive approach generated themes. Data were coded by one main coder (JD) but to increase reliability of analysis SU/C focus groups data were also coded by CM and a sample of staff focus group data was coded by NJ. Coding was sentence by sentence to allow accurate comparison. Differences in coding were discussed and where necessary codes were re-defined and the process repeated. On

For peer review only - http://bmjopen.bmj.com/site/about/guidelines.xhtml

the second occasion over 90% agreement was reached on codes allocated. Coding was according to the three areas of interest, value and unintended consequences, concerns and implementation. Data saturation was achieved.

Results

Characteristics of the sample

SU/C groups in NHS trusts A and B involved six and five participants respectively. The composition of the staff groups was according to Table 1; junior doctor refers to doctors in their first (FY1) or second (FY2) year post qualification or registrar which is the first promotion post qualifying. Senior doctor refers to senior registrar which is a pre-consultant grade and consultant, the most senior medical person. Grades of nurses include below six (grade five being the most junior post qualification nurse) and above six (grades 7 charge nurse, grade 8 matron and above nurse specialists or clinical managers). Allied professionals included physio and occupational therapists. We did not formally ask SU/Cs about the nature of their or their friend/relative's hospital stay, however, examples given to support views offered suggests a wide range of experiences for example, heart bypass surgery, caring for those at the end of life and experiencing hospital admission with a chest infection and subsequent pneumonia. SU/Cs talked about admissions to the Emergency Department, Intensive Care Unit and both medical and surgical wards. There were eight staff focus groups with the number of participants ranging from two to 16 across both Trusts. The duration of focus groups ranged between 22 minutes and one hour 29 minutes.

Figure 3: Staff Focus Group Participants

Overall Findings

There were eleven themes arranged according to the aims of the study, "value and unintended consequences", "concerns" and "implementation" represented in figure 4 which are elaborated below with verbatim quotes.

Figure 4: Themes resulting from data analysis according to the study aims

Value and unintended consequences

Decision-making

Staff talked about the potential value of using CARS as a decision aid for choosing active or supportive care or for "do not resuscitate" decisions.

For peer review only - http://bmjopen.bmj.com/site/about/guidelines.xhtml

“ . . . on admission . . .might help triage” Sr Dr FG1

“. . . decisions about end of life care as well; guide DNR [do not resuscitate] *decision making”* Sr Dr, FG1

This was considered within different *contexts* of care; in some areas a high score might suggest supportive care (e.g. general medical areas) and in other areas (e.g. paediatrics) it would be expected even the smallest chance of survival suggests active care.

“some clinicians would say even half a percent chance of survival is enough. . . . where do we draw the line?” Sr Dr, FG1

Staff considered the score would give them extra confidence in making clinical decisions.

“Having the CARS [Computer Aided Risk] *score then makes you think no ok I am on the right way and it gives you a bit more confidence*” Sr N, FG3

SU/C groups were largely positive about its potential to play a useful role in improving patient safety because it could challenge clinician’s preconceived ideas and provide additional information for clinicians to make a judgement.

“what I’ve seen is that they get this idea in their head of what's wrong with you and this is perhaps a good way of making double check whether it’s that preconceived idea” SU/C Trust B

SU/Cs suggested staff might need help in picking-up on changes in a patient’s condition, particularly where their specialism is not end-of-life care but something where mortality risk is not as great.

“Because he’s under pressure he’s going to treat the thing that he’s the expert in and he’s been called down to A&E to deal with, he’s not a GP [general practitioner] *is he? He’s not a full spectrum doctor is he; he’s a specialist.”* SU/C Trust B

SU/Cs felt this trigger could add weight to a patient’s or carer’s expressed concerns.

“probably get more action by having the score reading than just by somebody saying to the nursing staff oh I don’t think my mother’s very well” SU/C Trust B

Clinical judgement

Staff were vocal in discussing the impact of the score on clinical judgement. Many thought it would increase confidence in decisions.

For peer review only - http://bmjopen.bmj.com/site/about/guidelines.xhtml

> *"I think CARS* [Computer Aided Risk] *score, well all scoring systems are useful anyway as they are a starting point to see how sick your patient is, it's a good way of just getting all the information in one go"* Sr Dr, FG3

Others expressed concerns their clinical judgement may be undermined by the score. Discussions focused on the appropriate "weight" to give the score in the clinical decision making process particularly when the score and their own judgement conflicted.

> *"We want the space to use that judgement . . ."* Jr Dr FG1

> *"With all of these things* [scores] *you stop taking a clinical interest in the patient and just look at the numbers" NS* FG2

SU/Cs recognised the potential of the score but were overwhelmingly cautious a score should never replace clinical judgement.

> *"as long as it's another helpful factor in deciding what to do as opposed to being the determining factor cause it would frighten me a lot if it was a determining factor"* SU/C Trust A

Litigation

Staff saw potential positive and negative elements to the score in terms of supporting their decisions or otherwise and the potential for litigation.

> *"If you need to back up your clinical judgement to the coroner* [the Computer Aided Risk Score] *would help I think"* Sr Dr FG1

> *"Someone is going to say, I am going to pore over those notes and find out why this person has died, whereas, they may not have done previously. Conversely this person had a high chance of dying, why did you carry on with your treatment which, you know, which was futile"* Sr DR FG1

Communication

Most staff considered the score would aid or prompt communication with patients about their prognosis. There was disagreement about whether or not it might be best to give the actual score or a description of the situation.

> *"We tend to use more descriptive terms rather than actual numbers . . . patients are appreciative of honesty"* Sr Dr FG1

For peer review only

SU/C participants talked at length about the score's potential role in assisting communication about a patient's condition to their family, helping them accept the seriousness of the condition when some find this difficult.

> *"If he had a score - today this is how bad she actually is its likely going to be soon, that would have helped him deal with the situation better"* SU/C Trust A

However, there was much debate amongst both staff and SU/Cs ion about whether or not patients and carers should be actively informed of the score.

> *"One of the biggest things in any hospital anywhere is communication and information they're not told, and I'm sure they would like to be told"* SU/C Trust B

> *"They* [staff] *are a little too tactful, little too polite, little too sensitive. It's a very sensitive situation but the score might help"* SU/C Trust B

But it was also argued this score was primarily for clinicians and whilst should not hidden, it need not be routinely provided in the same format for everyone.

> *"I think if the family are told they are gravely ill that would be more human than they are an eight point four"* SR Dr FG2

Components of the algorithm

Staff discussed at length the need to know the component parts of the score and access the latest contributing values.

> *"I would like the people who are reviewing the score to be able to understand it properly. Otherwise you will get people who are becoming overly worried about it when they don't actually, can't interpret it and don't understand it."* Jr Dr FG5

Accuracy

There was some discussion of when the risk presented by the CARS may or may not be accurate. Frequent examples offered of where CARS may not be accurate were COPD, CKD, terminal conditions and congenital diseases.

> *"What about COPD* [Chronic Obstructive Pulmonary Disease] *. . . at the same time people with significant co-morbidities will have a worse outcome?"* Jr Dr FG1

SU/C groups were interested in exploring the accuracy of the score:

> *"It needs to be sensitive both ways . . . otherwise everybody will be in the high dependency unit"* SU/C Trust B

For peer review only - http://bmjopen.bmj.com/site/about/guidelines.xhtml

Resource Implications

Staff raised questions about the potential resource needs associated with CARS e.g. intensive Care Unit beds and blood tests (it was speculated these would become more frequent).

> *"Can I just ask what sort of impact this will have on the labs?"* NS FG2

> *"the extra expenditure. . . you have an ethical dilemma because you have a patient who's got a score you've gone to escalating to HDU* [High Dependency Unit], *ICU* [Intensive Care Unit], *high observations units."* NS FG2

SU/C groups were also concerned about resources but this focused on extra workload for staff. They were also worried the score would mean less face-to-face care.

> *"My concerns would be they're already under extreme pressure, if this is going to be another assessment that they have got to carry out on patients that's increasing the pressure at a time when they're already stressed out"* SU/C Trust B

Implementation

Presentation

Staff focus groups had ideas as to the presentation of CARS.

> *"If I were using it myself as a physician I would want a specific percentage"* Jr Dr FG1

They emphasised the need to see the trend in the score.

> *"Putting it all together in a score is helpful when you are the person on call who doesn't know them and you can see the trend of that score and it's helpful."* Jr Dr FG5

> *"It would be useful to see it as a graph* [trend]" Jr Ns FG8

When discussing presentation of the score the SU/Cs focused mainly on the communication of it by staff (reported above) and whether they should have direct access/sight of the score.

> *"To see it change in front of your eyes that might be even more terrifying"* SU/C Trust A

CARS compared with NEWS

Staff appreciated CARS was more sensitive than NEWS, and though they appreciated, unlike NEWS, the CARS was a complex statistical equation, not possible to calculate by hand, they were keen to see it.

> *"Can you copy that algorithm? Can we have a look at that?"* NS FG2 Trust B

For peer review only - http://bmjopen.bmj.com/site/about/guidelines.xhtml

The key issue with respect to this comparison was the potential for CARS to suggest one action should be taken and NEWS suggests another (e.g. one indicates escalation the other does not).

> *"NEWS* [National Early Warning Score] *score high and CARS low or vice versa therefore we've then got a confusion to the people who are actually on the shop floor where one thing is telling them to do this escalation and the other is saying you don't need to that is another reason I would be worried about it"* O FG2

Staff considered the comparative utility of NEWS and CARS with a particular focus on whether blood tests would delay a calculation of the score, or, whether CARS would be updated when any new data item (e.g. NEWS components such as temperature/pulse) became available.

> "[CARS unlike NEWS] *it might take three or four hours .. .if it relies on blood tests"* Sr Dr FG1

Finally, there was indication staff wanted to see the score demonstrated to be effective in relation to *people* in addition to being mathematically valid.

Guidelines

Staff discussed the specific procedures for CARS' role in confirming or support clinical judgement:

> *"I don't think you can use the CARS score as a trigger to make any specific clinical action, it is an alert that there may be a clinical problem there, there is a clinical problem there and then you need to find out what it actually is, it may be you need to look more closely at the biochemistry or whatever, whereas, the NEWS score is more specific"* NS FG2

They initially suggested the need for an escalation protocol or guide (where actions are prescribed according to score).

> *". . . with the NEWS* [National Early Warning Score] *if you have a score of five an above obviously that is an escalation process whereas with the CARS we don't know"* NS FG2

When vignettes were brought in during the second staff focus groups, staff were less likely to feel the need for an escalation protocol or a guide.

> *"I'd be happy to make that decision, because I would have already made that decision without the CARS score but it would just help support that decision making"* Jr Dr FG3

> *"I don't think it* [CARS] *changes the clinical management because the clinical management is always going to be based on the individual in front of you with their individual bloods and things. Putting it all together in a score is helpful"* Jr Dr FG5

Where staff wanted guidance this was sometimes to protect against *criticism* about inappropriate response to a high CARS

For peer review only - http://bmjopen.bmj.com/site/about/guidelines.xhtml

"If there isn't [a protocol]*, you go to the doctor and say the CARS has come up at this score, and they say yes that's because. . . . . they would always have a rationale . . . but at least you are covering yourself by saying this CARS scores this, and you are acting on it"* Sr Ns FG7

This was linked to concerns about litigation.

"Then one day someone will turn around and say but the CARS score was 10 and you didn't do this, so I think that's just the world we live in and we have to have a clear role when we introduce it or otherwise. . . ." Jr Dr FG 4

Some felt the guidance would ensure (insist upon) a response from a senior clinician.

"You can say to the doctor look I am just following the protocol" Jr Ns FG4

Others suggested it would serve as support for more junior staff.

"Junior medical & nursing staff . . . I would worry that they would, that they might lack clinical prowess" Sr Dr FG3

Strategy

There was a lot of discussion about a strategy for implementation. One group (FG2) spent a lot of time discussing the need for a "champion". This related to their experience with NEWS. The suggestion was one of the outreach team would be best placed for this. Discussion took place about the extent of education required and there was a consensus, although the intention was to implement CARS in medical wards only, staff movement between departments was such, a hospital wide strategy would be appropriate.

"We need people to champion this" O FG2

"The big nooks and crooks is going to be education, needs training, information as well. If you're just looking at one area, there are medical people coming through that area teams to you need to target them all." Sr Dr FG2

Discussion

Our approach in developing a novel automated CARS has been a collaboration with front line staff and SU/Cs as part of the project team as well as participants. We conducted 8 focus groups with 11 service users and carers and 45 health care practitioners in two NHS acute hospitals.

We found participants from staff and SU/C groups were interested in the development of the CARS score and appreciated such efforts being made to improve patient safety at their hospital sites. Staff and SU/Cs recognised the potential of CARS but were clear the score should not replace or undermine clinical judgments. Staff recognised CARS could enhance clinical decision making/judgments and aid

For peer review only - http://bmjopen.bmj.com/site/about/guidelines.xhtml

communication with patients. They wanted to understand the components of CARS and be reassured about its accuracy but were concerned about the impact on intensive care and blood tests. Staff preferred CARS to be shown as a score (without descriptive labels) graphed over time to monitor changes. The need to clarify how CARS and NEWS would work alongside each other especially where they were divergent was highlighted. SU/C has mixed views about the extent to the score should be shared with patients.

As far as we are aware previous studies on the design, development and implementation of risk scores have not reported on the views of staff and SU/C, so we are unable to determine the extent to our implementation of these risk scoring systems into routine clinical practice requires careful consideration of the views of staff and patients. However, our purposive sampling and data saturation suggests our findings with regard to the value, unintended consequences and concerns related to CARS may be generalizable to the implementation of risk scores elsewhere. Furthermore the themes identified highlight risk scores are complex interventions being introduced into complex adaptive systems and the voice of staff and SU/C is an important element of co-design to maximise the success of risk scores. In our case, the voice of staff and SU/C was an integral and iterative part of the design of CARS and has led to some important insights and design changes including the following: (1) CARS will update over time and be available as a graph with all its subcomponents also being shown at the same time. (2) The relationship of CARS with NEWS was important to clarify. We have now designed our risk score to use NEWS in the first instance and then incorporate blood test results as an when available. About ¼ of patients do not have a blood test results. (3) We have decided to present the score as a % (0 to 100) without any descriptive labels (e.g. low/medium/high). (4) The score will be visible on the electronic patient record but will not be a "pop-up" alert. We are now working with both NHS trusts and taking a staged approach to implementing CARS as a quality improvement programme with iterative development. We continue to involve staff and SU/Cs in this process. The qualitative work reported here continues to map the process, identify early problems and support solutions.

The process of involvement of stakeholders within interventions is challenging and we can usefully reflect on the limitations of our effort in this regard. For staff, finding adequate time for their quality input was hard and, whilst we achieved this through flexible approaches to focus group recruitment, venues and timings, the process revealed warnings about the "unfinished business" and "unanswered questions" staff still have about implementation which would benefit from further involvement. With respect to SU/Cs, we can refer to notions of an involvement hierarchy of "consultation, collaboration, and user-control" (e.g. Hanley et al 2004) to critique our own approach. We predominantly consulted via our focus groups. Whilst we extended this to "collaboration" with our steering group members

For peer review only - http://bmjopen.bmj.com/site/about/guidelines.xhtml

who maintained input throughout, this was limited in frequency, in part because of the practicalities of bringing busy people together. Much of the implementation and research therefore was undertaken on at individual sites, integrated into daily hospital working, mirroring iterative quality improvement process. Including SU/Cs in this is fraught with difficulty and is currently rare in healthcare (Green et al 2018). Nevertheless as healthcare is increasingly using computer aided decision support systems are seen a key to achieving major gains in quality and patient safety (Baker, 2001), we urge some sort of co-design approach is necessary to maximise the successful implementation and evaluation of risk scores in healthcare.

Conclusions

Staff and patients have important, often complex, insights to support the development and implementation of CARS need to be addressed if CARS is to be successfully used in routine practice. We are now working with both NHS trusts and taking a staged approach to implementing CARS with continual feedback from staff and SU/Cs in this process.

For peer review only

For peer review only - http://bmjopen.bmj.com/site/about/guidelines.xhtml

Contributorship

MAM and DR had the original idea for this work. NJ was the study coordinator. JD, CM and NJ were the leads for the qualitative study. JD, NJ, CM and MAM wrote the first draft of this paper. All authors subsequently assisted in redrafting and have approved the final version.

Data Sharing

No additional data available.

Competing Interests

The authors declare funding from the bodies listed below.

Acknowledgements

This study is funded by the Health Foundation and the National Institute for Health Research (NIHR) Yorkshire and Humber Patient Safety Translational Research Centre (NIHR Yorkshire and Humber PSTRC).

The views expressed in this article are those of the author(s) and not necessarily those of the Health Foundation, the NHS, the NIHR, or the Department of Health and Social Care.

The Health Foundation is an independent charity working to improve the quality of health care in the UK.

We acknowledge the vital role of the Service User and Carer Involvement in Research Group who not only contributed to the project steer but also contributed practically to the planning and execution of this qualitative work.

We offer thanks to our SU/C and practitioner participants.

We acknowledge the wider project team for their contributions to the design of the work and recruitment of participants.

For peer review only

For peer review only - http://bmjopen.bmj.com/site/about/guidelines.xhtml

References

Baker, A., 2001. Crossing the quality chasm: a new health system for the 21st century. *BMJ: British Medical Journal*, *323*(7322), p.1192.

Brabrand, M., Folkestad, L., Clausen, N.G., Knudsen, T. and Hallas, J., 2010. Risk scoring systems for adults admitted to the emergency department: a systematic review. *Scandinavian journal of trauma, resuscitation and emergency medicine*, *18*(1), p.8.

Braun, V. and Clarke, V., 2006. Using thematic analysis in psychology. *Qualitative research in psychology*, *3*(2), pp.77-101.

Department of Health, 2013, National Early Warning Score, National Clinical Guideline No. 1, https://health.gov.ie/wp-content/uploads/2015/01/NEWSFull-ReportAugust2014.pdf, last accessed July 2018

Faisal, M., Scally, A., Jackson, N., Richardson, D., Beatson, K., Howes, R., Speed, K., Menon, M., Daws, J., Dyson, J., Marsh, C., Mohammed, M.A., Development of a novel computer aided risk score to predict the risk of death in hospital for acutely ill medical patients using their first blood test results and vital signs after admission, *BMJ Open*, in press.

Green SA, Carnegie W, Barber S, Matthew D, Matthews R. Co-designing within quality improvement initiatives: Notes from the field. JHD. 2018;3(1):49–56. https://doi.org/10.21853/JHD.2018.3

Guest, G., Bunce, A. and Johnson, L., 2006. How many interviews are enough? An experiment with data saturation and variability. *Field methods*, *18*(1), pp.59-82.

Hands, C., Reid, E., Meredith, P., Smith, G.B., Prytherch, D.R., Schmidt, P.E. and Featherstone, P.I., 2013. Patterns in the recording of vital signs and early warning scores: compliance with a clinical escalation protocol. *BMJ quality & safety,* **22**(9), pp. 719-726.

Hanley B, Bradburn K, Barnes M, Evans C, Goodare H, Kelson M, Kent A, Oliver S, Thomas S & Wallcraft J (2004) Involving the Public in NHS, Public Health and Social Care Research: Brieifing Notes for Researchers, 2nd Edn. INVOLVE. Hampshire.

For peer review only

For peer review only - http://bmjopen.bmj.com/site/about/guidelines.xhtml

Figure 1: Focus group questions round 1

Staff focus group questions	Service User/Carer focus group questions
**General:** What are your thoughts on the CARS? How might the score be valuable? How might the score present challenges? Can you see any problems implementing the score in practice?<br>**Knowledge related to and presentation of the score:** What things do you still need to know about the CARS? What information do you think health care practitioners will need to use the score? How do you think this (information/score) is best disseminated to health care practitioners? How much weight might be attributed to the score in terms of clinical judgement? Talk us through how this might work. How might the score combine with clinical examination and patient reported symptoms?<br>**Components of the score:** Would you share your thoughts how accurate (reliable/valid) you consider the CARS to be based on the information/presentation we have given?<br>**Your responses to CARS:** Talk us through the likely process of action from receiving the CARS. How might the score might aid practitioners' decisions about treatment and care? Can you think of any circumstances where the score might support or undermine your clinical decision? Would you share the CARS score with patients? Relatives and carers? Are there any resource implications associated with the CARS?<br>**Organisational matters:** How might the CARS be used by the institution with regard to resource management? Is there any value to having score recorded in medical records? Any problems?	**General:** What are your thoughts and feelings about the CARS? How might the score be valuable? Do you have any worries about the score?<br>**Awareness:** What information about the score generally do you think people would like? What are your thoughts on patients being told about the score? What about relatives and carers?<br>**Impact on care:** The proposition is that the score might aid practitioners in treatment and care choices (e.g. admit/discharge home, where to admit, active treatment, supportive care)? How does this proposition sound to you? Does this proposition raise any concerns for you? How do you see the patient and carers role in this might be?<br>**Organisational matters:** How might the CARS be impact on resources/resource management or organisation of care?<br>**The following sentences were copied onto cards and presented in turn**<br>In response to the CARS practitioners may. . . . .<br>In response to the CARS I (as a patient)/patients may . . . .<br>In response to the CARS I (as SU/C)/my family . . . . . .<br>The CARS may be valuable for. . . . .<br>The problems with the CARS might be. . . . . .<br>If I knew my/my relative's CARS I might feel . . . .<br>The value for practitioners with regard to the CARS might be. . . . .<br>The problems for practitioners with regard to CARS might be. . . . .<br>Practitioners should share the score with patients/carers when . . . .<br>The best way to communicate the score might be. . . . . . .<br>Putting a number on my risk of deterioration is . . . .

For peer review only

Figure 2: Focus group content round 2

Vignette example

Patient Age (years): [85], Gender: [Male], time of admission: [17:25]
Mode of presentation: [Acute/Emergency]

Description of presentation

Recent previous admission with AKI[1] and CKD[2], admitted as wife not coping – patient doubly incontinent with very poor mobility and new diarrhoea, past medical history of dementia, T2DM[3], previous stroke and osteoarthritis, on examination abdominal soft and non-tender, chest clear, heart sounds normal, commenced on IV[4] fluids

Time of Index NEWS: 00:50 NEWS [1]
Respiratory Rate: [18] breaths per minute
Oxygen Saturations [97] %
Temperature [35.1] degrees Celsius
Systolic BP [150] mmHg
Diastolic BP [65] mmHg
Pulse Rate [75] beats per minute
Level of consciousness [Alert]

Index blood test results

Blood Test	Result
Albumin (g/L)	[35]
Creatinine (umol/L)	[986]
Haemoglobin (g/dl)	[103]
Potassium (mmol/L)	[5.2]
Sodium (mmol/L)	[142]
Urea (mmol/L)	[38.2]
White blood cell count (10^9 cells/L)	[5.5]
Platelets (10^3/microliter)	[281]

Acute Kidney Injury Score [3]

Focus group questions

Response to Vignette: Talk us through your decisions regarding the vignette (use up to 3 vignettes per group).
Response to CARS: The CARS score for this patient is [present score]. What are your thoughts on CARS given this context? Would the score change the decisions you made about treatment and care? How much would you attribute to the score in terms of clinical judgement? How might the score combine with clinical examination and patient reported symptoms?
Knowledge related to the score: How do you think this CARS is best implemented?
Presentation of the score: What did you think to the way we presented the score (scale of 1 to 8 with descriptors such as low, low, moderate and high)? How could it be better presented?
Finally, visual mock-ups of the score were presented and comments requested.

[1] Acute Kidney Injury [2] Chronic Kidney Disease [3]Type 2 Diabetes Mellitus [4]Intravenous

For peer review only

Figure 3: Staff Focus Group Participants

Practitioner	Round 1		Round 2						Total
	A[1]	B[2]	A[3]	A[4]	A[5]	B[6]	B[7]	B[8]	
	n=7	n=10	n=16	n=2	n=3	n=3	n=2	n=2	n=45
Doctor (Dr)	5	2	6	0	3	1	0	0	17
(Senior (Sr) doctor (Dr) consultant/senior registrar)	(3)	(2)	(6)	(0)	(0)	(1)	(0)	(0)	(12)
(Junior doctor (Jr) registrar, FY2, FY1)	(2)	(0)	(0)	(0)	(3)	(0)	(0)	(0)	(5)
Ward based Nurse (N)	0	2	6	2	0	1	2	2	15
Senior (Sr) Nurse (N) (above band 6)	(0)	(2)	(5)	(2)	(0)	(0)	(2)	(0)	(11)
Junior (Jr) Nurse (N) (below band 6)	(0)	(0)	(1)	(0)	(0)	(1)	(0)	(2)	(4)
Nurse Specialist (NS)	2	5	0	0	0	0	0	0	7
Health Care Assistant (HCA)	0	0	2	0	0	0	0	0	2
Other (O) (allied professionals)	0	1	2	0	0	1	0	0	4

NB: A/B is Trust A/B. [] is the count of number of focus groups. n= is the number of participants.

For peer review only

Figure 4: Themes resulting from data analysis according to the study aims

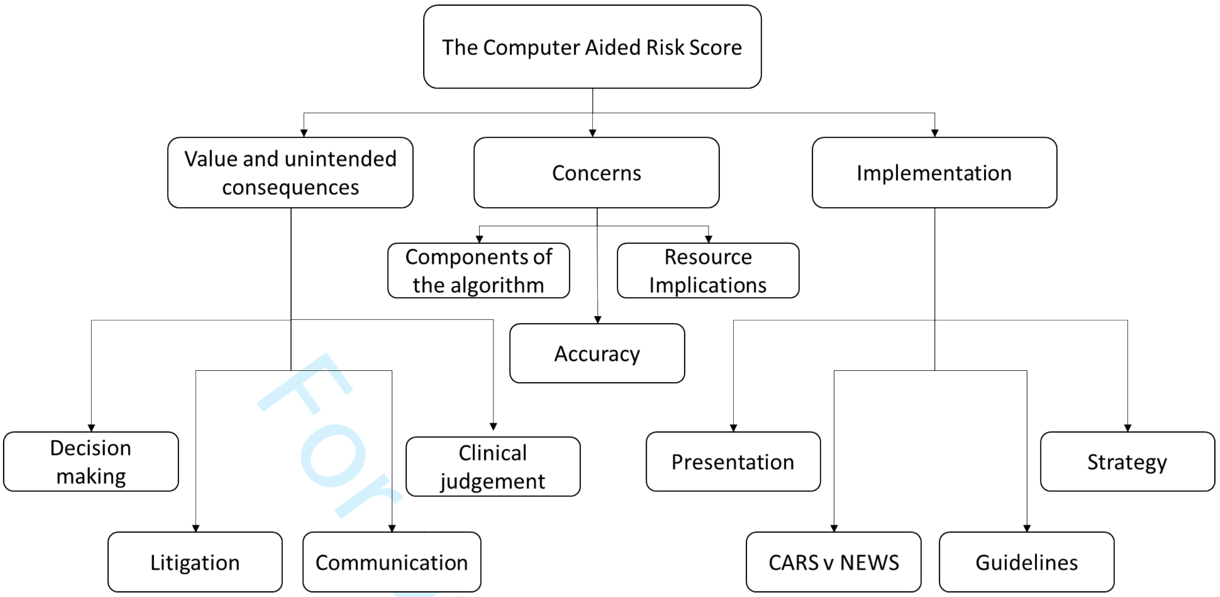

For peer review only

BMJ Open

Understanding and applying practitioner and patient views on the implementation of a novel automated Computer Aided Risk Score (CARS) predicting the risk of death following emergency medical admission to hospital: A qualitative study

Journal:	*BMJ Open*
Manuscript ID	bmjopen-2018-026591.R1
Article Type:	Research
Date Submitted by the Author:	31-Jan-2019
Complete List of Authors:	Dyson, Judith; University of Hull, Health and Social Work<br>Marsh, Claire; Bradord Institute for Health Research, Quality and Safety<br>Jackson , Natalie ; Bradord Institute for Health Research, Quality and Safety<br>Richardson, Donald; York Teaching Hospital NHS Foundation Trust Hospital, Renal Medicine<br>Faisal, Muhammad; University of Bradford, Faculty of Health Studies; University of Bradford<br>Scally, Andrew ; University of Bradford, School of Health Studies<br>Mohammed, Mohammed ; University of Bradford, School of Health Studies
**Primary Subject Heading**:	Qualitative research
Secondary Subject Heading:	Evidence based practice, Health services research
Keywords:	Computer Aided Risk Score, Implementation, Service User Involvement, CARS, Early warning score, Hospital Mortality

SCHOLARONE™
Manuscripts

Understanding and applying practitioner and patient views on the implementation of a novel automated Computer Aided Risk Score (CARS) predicting the risk of death following emergency medical admission to hospital: A qualitative study

Dr Judith Dyson, University of Hull, Cottingham Road, HULL, UK HU6 7RX, T: 01482 463806, E: j.dyson@hull.ac.uk
Dr Claire Marsh, Bradford Institute of Health Research, Bradford UK
Ms Natalie Jackson, Bradford Institute of Health Research, Bradford UK
Dr Donald Richardson, York Teaching Hospitals NHS Foundation Trust, York UK
Dr Muhammad Faisal, University of Bradford, Bradford, UK
Dr Andrew Scally, University of Bradford, Bradford, UK
Prof Mohammed A Mohammed, University of Bradford, Bradford, UK

Word count 3997

For peer review only

For peer review only - http://bmjopen.bmj.com/site/about/guidelines.xhtml

Understanding and applying practitioner and patient views on the implementation of a novel automated Computer Aided Risk Score (CARS) predicting the risk of death following emergency medical admission to hospital: A qualitative study

Abstract (281 words)

Objectives: The Computer Aided Risk Score (CARS) estimates the risk of death following emergency admission to medical wards using routinely collected vital signs and blood test data. Our aim was to o elicit the views of health care practitioners (staff) and Service Users and Carers (SU/C) on i) the potential value, unintended consequences and concerns associated with CARS and practitioner views on ii) the issues to consider before embedding CARS into routine practice.

Setting: This study was conducted in two NHS Hospital Trusts in the North of England. Both had in-house IT development teams, mature IT infrastructure with electronic National Early Warning Score (NEWS) and were capable of integrating NEWS with blood test results. The study focused on emergency medical and elderly admissions units. There were 60 and 39 acute medical/elderly admissions beds at the two NHS hospital Trusts.

Participants: We conducted eight focus groups with 45 health care practitioners and two with 11 service users and carers (SU/Cs) in two NHS acute hospitals.

Results: Staff and SU/Cs recognised the potential of CARS but were clear the score should not replace or undermine clinical judgments. Staff recognised CARS could enhance clinical decision making/judgments and aid communication with patients. They wanted to understand the components of CARS and be reassured about its accuracy but were concerned about the impact on intensive care and blood tests.

Conclusion: Risk scores are widely used in healthcare, but their development and implementation does not usually involve input from practitioners and SU/Cs. We contributed to the development of CARS by eliciting views of staff and SU/Cs who provided important, often complex, insights to support the development and implementation of CARS to ensure successful implementation in routine clinical practice.

For peer review only - http://bmjopen.bmj.com/site/about/guidelines.xhtml

Article Summary

- This paper reports the involvement of practice staff and service users/carers in considering the implementation of the Computer Aided Risk Score (CARS) into clinical practice
- Staff and service users/carers have offered important, complex insights which could support the implementation of risk scores into clinical practice
- Both practitioners and SU/Cs identified potential value and unintended consequences of the score in practice

Strengths and limitations

- Our research takes a rare approach of including healthcare practitioner and service user/carer (SU/C) involvement in the development of a risk score
- Finding adequate time for practitioner input was hard and needed flexible approaches to focus group recruitment, venues and timings

For peer review only

For peer review only - http://bmjopen.bmj.com/site/about/guidelines.xhtml

Understanding and applying practitioner and patient views on the implementation of a novel automated Computer Aided Risk Score (CARS) predicting the risk of death following emergency medical admission to hospital: A qualitative study

Dr Judith Dyson, University of Hull, Cottingham Road, HULL, UK HU6 7RX, T: 01482 463806, E: j.dyson@hull.ac.uk
Dr Claire Marsh, Bradford Institute of Health Research, Bradford UK
Ms Natalie Jackson, Bradford Institute of Health Research, Bradford UK
Dr Donald Richardson, York Teaching Hospitals NHS Foundation Trust, York UK
Dr Muhammad Faisal, University of Bradford, Bradford, UK
Dr Andrew Scally, University of Bradford, Bradford, UK
Prof Mohammed A Mohammed, University of Bradford, Bradford, UK

Key Terms: "Computer Aided Risk Score", CARS, "Service User Involvement", Implementation, "Early Warning Score", "Hospital Mortality"

Word count: 3997

For peer review only

Introduction

A UK wide study of 10 hospitals estimated 5% of deaths were preventable and 30% of these were attributable to poor clinical monitoring [1]. If risk of death information was available to clinical staff it was likely to enhance patient safety [2], however, there are no established risk equations for acutely admitted medical patients. Furthermore, while several studies have considered the use of physiological signs *or* blood tests in the assessment of patient few consider combining the two [2]. Although NEWS is known to predict mortality in the hospital and pre-hospital setting [3] it is not suitable for some groups of patients [4]. This research team therefore developed a novel computer aided risk score (CARS) for estimating the risk of in-hospital mortality following emergency medical admission to hospital [5] in two hospitals. CARS was designed to rely on variables already routinely collected and electronically recorded as part of the process of care including vital signs data (based on a National Early Warning Score (NEWS))[6] and blood test results [7]. CARS demonstrated better discrimination and calibration than blood tests and NEWS separately [5].

Despite the widespread use of risk scores to enhance decision making in healthcare, as identified by Braband et al [2], there is little or no documentary evidence of the involvement of healthcare practitioners and service users or carers (SU/Cs) in the design, development and implementation of this type of risk score. Our research responds to this gap.

For peer review only - http://bmjopen.bmj.com/site/about/guidelines.xhtml

Concurrent to the statistical modelling work, we conducted focus groups with healthcare practitioners and SU/Cs to feed into the on-going development of CARS. The aims of the focus groups were to establish i) health care practitioner (hereafter "staff") and SU/C views on the potential value, unintended consequences and concerns associated with the development of the CARS and ii) staff views on how CARS should be adopted in practice/implementation needs

Methods

Ethical approval

Ethical approval was granted from National Research Ethics Service (15/YH/0348) and Research Governance approval from the trusts involved in the study. All participants gave signed consent after receiving written information about the study.

Patient and Public Involvement

The "Service User and Carer Involvement in Research Group" (SURG) at the University of Bradford supported the project as members of the project steering group and as a focus group advisory team. Their contribution included co-design of project materials, support of the methodology (e.g. recruitment strategies) and offering comments and suggestions based on data gathered.

Participants

This study was conducted in two NHS Hospital Trusts in the North of England (referred to hereafter as Trust A and Trust B). Both had in-house IT development teams, mature IT infrastructure with electronic NEWS and were capable of integrating NEWS with blood test results. The study focused on emergency medical and elderly admissions units. There were 60 and 39 acute medical/elderly admissions beds at Trusts A and B respectively.

SU/Cs were competent adults (aged over 18 years) who were members of the public, who had either been in hospital themselves any time in their adult life, or who had experienced a relative in hospital. Staff were, any practitioner working in areas where we intended to implement CARS (acute assessment units, medical wards and older person in-patient units) or acute outreach staff (nurse or doctor called upon to offer advanced assessment and input), were eligible. Due to the additional aim of the staff groups (implementation needs) we held separate SU/C and staff focus groups.

For peer review only - http://bmjopen.bmj.com/site/about/guidelines.xhtml

Design

Ten focus groups were held over two rounds in each Trust (eight staff groups and two SU/C groups). A first round of staff and SU/C focus groups was conducted at the beginning of CARS development and commenced with a brief presentation about CARS, its rationale and development, then asked participants for their thoughts, feelings and concerns in relation to implementation of a CARS at their hospitals. Focus group schedules were informed by the literature relating to other risk scores (e.g. NEWS). Results from focus groups were fed back to the CARS research team who then further developed CARS and its implementation package (Figure 1 illustrates round one focus group questions). Subsequently, a second round of focus groups with staff explored CARS implementation needs in greater depth. After a presentation about CARS, vignettes were used (developed from case note reviews), to allow staff to understand how CARS scores relate to real clinical scenarios (Figure 2 offers an example of a vignette and round two focus group questions). It was our intention to run groups of between 6 and 12 participants. Due to the challenges of staff time and availability, this was revised to smaller group sizes for the second staff focus groups. JD led staff focus groups, CM led SU/C groups and NJ supported all groups (none of whom had a previous relationship with participants and all were experienced in running focus groups).

Figure 1: Focus group questions round 1

Figure 2: Focus group content round 2

Procedure

CM approached the Patient Experience leads at each hospital as a gatekeeper for recruitment to SU/Cs. Patient Experience leads contacted members of their forums through email, posters and verbal invites. Interested people contacted CM directly and were given participant information sheets before deciding to attend. Clinical partners from the CARS implementation teams at both hospitals introduced JD and NJ (electronically or in person) to the nurse in charge of relevant hospital areas. Charge nurses circulated email invitations to qualified medical and nursing staff of all grades with participant information. They contacted the research team if they were interested in participating. All interested SU/C and staff were included.

The first round of focus groups (SU/C and staff) took place over a six-month period from May 2016 and the second staff focus groups occurred between May and July 2017. For staff, careful recruitment identified a diverse range of participants in terms of their professional role and experience. There were slightly more medical staff, which was expected given the relevance of the score. We expected

For peer review only - http://bmjopen.bmj.com/site/about/guidelines.xhtml

focus groups including 25 participants within groups would be enough to achieve data saturation [8]. With one exception, all focus groups took place in hospital meeting rooms or offices. The exception was one staff focus group, which took place at a conference centre (for the convenience of staff).

Analysis

Focus groups were audio recorded and transcribed verbatim and transcripts imported into NVivo v.11 data management software. Data were subject to thematic analysis [9]. An inductive approach generated themes. Data were coded by one main coder (JD) but to increase reliability of analysis SU/C focus groups data were also coded by CM and a sample of staff focus group data coded by NJ. Coding was sentence by sentence to allow accurate comparison. Differences in coding were discussed and where necessary codes were re-defined and the process repeated. On the second occasion over 90% agreement was reached on codes allocated. Coding was according to the three areas of interest, value and unintended consequences, concerns and implementation. Data saturation was achieved; no new codes were derived from data from the last two focus groups.

Results

Characteristics of the sample

SU/C groups in NHS trusts A and B involved six and five participants respectively. The composition of the staff groups was according to figure 3; junior doctor refers to doctors in their first (FY1) or second (FY2) year post qualification or registrar (first promotion post qualifying). Senior doctor refers to senior registrar (pre-consultant grade) and consultant (most senior medical person). Grades of nurses include below six (five being the most junior post qualification nurse) and above six (seven charge nurse, eight matron and above nurse specialists or clinical managers). Allied professionals included physio and occupational therapists. We did not formally ask SU/Cs about the nature of their or their friend/relative's hospital stay, however, examples given to support views offered suggests a wide range of experiences (e.g. heart bypass surgery, caring at the end of life, chest infection and subsequent pneumonia). SU/Cs talked about admissions to the Emergency Department, Intensive Care Unit and both medical and surgical wards. There were eight staff focus groups with the number of participants ranging from two to 16 across both Trusts. There were six and five participants in SU/C groups in trusts A and B respectively. All participants contributed to focus groups. The duration of focus groups ranged between 22 minutes and one hour 29 minutes, mean duration 57 minutes.

Figure 3: Staff Focus Group Participants

Overall Findings

For peer review only - http://bmjopen.bmj.com/site/about/guidelines.xhtml

There were nine themes arranged according to the aims of the study, “value and unintended consequences”, “concerns” and “implementation” represented in figure 4 and elaborated below with verbatim quotes from a broad range of participants.

Figure 4: Themes resulting from data analysis according to the study aims

Value and unintended consequences

Decision-making and clinical judgement

Staff talked about the value of using CARS as a decision aid for choosing active or supportive care or for “do not resuscitate” decisions.

“ . . . on admission . . .might help triage” Sr Dr1 FG1

“. . . decisions about end of life care as well; guide DNR [do not resuscitate] *decision making”* Sr Dr3, FG1

This was considered within different *contexts* of care; in some areas a high score might suggest supportive care (e.g. general medical areas) and in other areas (e.g. paediatrics) it would be expected even the smallest chance of survival suggests active care.

“some clinicians would say even half a percent chance of survival is enough. . . . where do we draw the line?” Sr Dr3, FG1

Staff considered the score would give them extra confidence in making clinical decisions.

“Having the CARS then makes you think no ok I am on the right way and it gives you a bit more confidence” Sr N1, FG3

“I think CARS score, well all scoring systems are useful anyway as they are a starting point to see how sick your patient is, it’s a good way of just getting all the information in one go” Sr Dr1, FG3

SU/C groups were similarly positive about its potential to play a role in improving patient safety because it could challenge clinician’s preconceived ideas and provide additional information for clinicians to make a judgement.

“what I’ve seen is that they get this idea in their head of what's wrong with you and this is perhaps a good way of making double check” SU/C Trust B

Both staff and SU/Cs expressed concerns clinical judgement may be undermined by the score. Staff discussions focused on the appropriate “weight” to give the score in the decision making process particularly when the score and their own judgement conflicted.

For peer review only - http://bmjopen.bmj.com/site/about/guidelines.xhtml

"We want the space to use that judgement . . ." Jr Dr1 FG1

"With all of these things [scores] *you stop taking a clinical interest in the patient and just look at the numbers"* NS1 FG2

"as long as it's another helpful factor in deciding what to do as opposed to being the determining factor" SU/C Trust A

Litigation

Staff saw potential positive and negative elements to the score in terms of supporting their decisions or otherwise and the potential for litigation.

"If you need to back up your clinical judgement to the coroner [the Computer Aided Risk Score] *would help I think"* Sr Dr2 FG1

"Someone is going to say, I am going to pore over those notes and find out why this person has died, whereas, they may not have done previously. Conversely this person had a high chance of dying, why did you carry on with your treatment which . . . was futile" Sr Dr1 FG1

Communication

Most staff considered the score would aid or prompt communication with patients about prognosis. There was disagreement about it might be best to give the actual score or a description of the situation.

"We tend to use more descriptive terms . . . patients are appreciative of honesty" Sr Dr1 FG1

"If he had a score, today this is how bad she actually is its likely going to be soon, that would have helped him deal with the situation better" SU/C Trust A

SU/C participants talked at length about the score's potential role in assisting communication about a patient's condition, helping them accept the seriousness of the condition. However, there was much debate amongst both staff and SU/Cs about whether or not patients and carers should be actively informed of the score.

"One of the biggest things in any hospital anywhere is communication and information they're not told, and I'm sure they would like to be told" SU/C Trust B

"They [staff] *are a little too tactful, little too polite, little too sensitive . . the score might help"* SU/C Trust B

It was argued this score was primarily for clinicians and whilst it should not hidden, it need not be routinely provided in the same format for everyone.

For peer review only - http://bmjopen.bmj.com/site/about/guidelines.xhtml

"I think if the family are told they are gravely ill that would be more human than they are an eight point four" SR Dr1 FG2

Components of the algorithm/accuracy

Staff discussed at length the need to know the component parts of the score and access the latest contributing values. They were keen to know when CARS may or may not be accurate. Frequent examples offered of where CARS may not be accurate were Chronic Obstructive Pulmonary Disease (COPD), Chronic Kidney Disease (CKD) terminal conditions and congenital diseases.

"I would like the people who are reviewing the score to be able to understand it properly. Otherwise you will get people who are becoming overly worried about it when they don't actually, can't interpret it and don't understand it." Jr Dr1 FG5

"What about COPD [Chronic Obstructive Pulmonary Disease] *. . . at the same time people with significant co-morbidities will have a worse outcome?"* Jr Dr2 FG1

SU/C groups were also interested in exploring the accuracy of the score:

"It needs to be sensitive both ways . . . otherwise everybody will be in the high dependency unit" SU/C Trust B

Resource Implications

Staff raised questions about the potential resource needs associated with CARS e.g. intensive Care Unit beds and blood tests.

"Can I just ask what sort of impact this will have on the labs?" NS2 FG2

"the extra expenditure. . . you have an ethical dilemma because you have a patient who's got a score you've gone to escalating to HDU [High Dependency Unit]*, ICU* [Intensive Care Unit]*, high observations units."* NS3 FG2

SU/C groups were also concerned about resources but this focused on extra workload and they were worried the score would mean less face-to-face care.

"My concerns would be they're already under extreme pressure, if this is going to be another assessment that they have got to carry out on patients that's increasing the pressure at a time when they're already stressed out" SU/C Trust B

Implementation

Presentation

Staff focus groups had ideas as to the presentation of CARS and the need to see the trend in the score.

"If I were using it myself as a physician I would want a specific percentage" Jr Dr1 FG1

For peer review only - http://bmjopen.bmj.com/site/about/guidelines.xhtml

"Putting it all together in a score is helpful when you are the person on call who doesn't know them and you can see the trend of that score and it's helpful." Jr Dr1 FG5

"It would be useful to see it as a graph [trend]" Jr Ns1 FG8

When discussing presentation of the score the SU/Cs focused mainly on the communication of it by staff (reported above) and whether they should have direct access/sight of the score.

"To see it change in front of your eyes that might be even more terrifying" SU/C Trust A

CARS compared with NEWS

Staff appreciated CARS was more sensitive than NEWS, and though they appreciated, unlike NEWS, the CARS was a complex statistical equation, not possible to calculate by hand, they were keen to see it.

"Can you copy that algorithm? Can we have a look at that?" NS3 FG2 Trust B

The key issue with respect to this comparison was the potential for CARS to suggest one action should be taken and NEWS suggests another (e.g. one indicates escalation the other does not).

"NEWS [National Early Warning Score] *score high and CARS low or vice versa therefore we've then got a confusion to the people who are actually on the shop floor where one thing is telling them to do this escalation and the other is saying you don't need to"* O FG2

Staff considered the comparative utility of NEWS and CARS with a particular focus on whether blood tests would delay a calculation of the score, or, whether CARS would be updated when any new data item (e.g. temperature/pulse) became available.

"[CARS unlike NEWS] *it might take three or four hours .. .if it relies on blood tests"* Sr Dr3 FG1

Finally, there was indication staff wanted to see the score demonstrated to be effective in relation to *people* in addition to being mathematically valid.

Guidelines

Staff discussed the specific procedures for CARS' role in confirming or support clinical judgement:

"I don't think you can use the CARS score as a trigger to make any specific clinical action, it is an alert that there may be a clinical problem there, there is a clinical problem there and then you need to find out what it actually is, it may be you need to look more closely at the biochemistry or whatever, whereas, the NEWS score is more specific" NS4 FG2

For peer review only - http://bmjopen.bmj.com/site/about/guidelines.xhtml

They initially suggested the need for an escalation protocol or guide (where actions are prescribed according to score).

> *“. . . with the NEWS* [National Early Warning Score] *if you have a score of five an above obviously that is an escalation process whereas with the CARS we don’t know”* NS1 FG2

When vignettes were brought in during the second staff focus groups, staff were less likely to feel the need for an escalation protocol or a guide.

> *“I don’t think it* [CARS] *changes the clinical management because the clinical management is always going to be based on the individual in front of you with their individual bloods and things. Putting it all together in a score is helpful”* Jr Dr2 FG5

Where staff wanted guidance this was sometimes to protect against *criticism* about inappropriate response to a high CARS.

> *“If there isn’t* [a protocol]*, you go to the doctor and say the CARS has come up at this score, and they say yes that’s because. . . . . they would always have a rationale . . . but at least you are covering yourself”* Sr Ns1 FG7

This was linked to concerns about litigation.

> *“Then one day someone will turn around and say but the CARS score was 10 and you didn’t do this, so I think that’s just the world we live in and we have to have a clear role when we introduce it or otherwise. . . .”* Jr1 Dr FG 5

Some felt the guidance would ensure (insist upon) a response from a senior clinician.

> *“You can say to the doctor look I am just following the protocol”* Sr Ns1 FG4

Others suggested it would serve as support for more junior staff.

> *“Junior medical and nursing staff . . . I would worry that they would, that they might lack clinical prowess”* Sr Dr3 FG3

Strategy

There was a lot of discussion about a strategy for implementation. One group (FG2) spent a lot of time discussing the need for a “champion”. This related to their experience with NEWS. The suggestion was one of the outreach team would be best placed for this. Discussion took place about the extent of education required and there was a consensus a hospital wide strategy would be appropriate.

> *“We need people to champion this”* O FG2

For peer review only - http://bmjopen.bmj.com/site/about/guidelines.xhtml

"The big nooks and crooks is going to be education, needs training, information as well. If you're just looking at one area, there are medical people coming through that area teams to you need to target them all." Sr Dr1 FG2

Discussion

Our approach in developing CARS has been co-working with front line staff and SU/Cs as part of the project team as well as participants. We conducted 10 focus groups with 11 SU/Cs and 45 health care practitioners in two NHS acute hospitals. Participants were interested in the development of the CARS score and appreciated such efforts to improve patient safety at their hospital. They recognised the potential of CARS but were clear the score should not replace or undermine clinical judgments. Staff recognised CARS could enhance clinical decision-making and aid communication. They wanted to understand the components of CARS and be reassured about its accuracy and were concerned about the impact on resources. Staff preferred CARS to be shown as a score (without descriptive labels) graphed by time to monitor changes. Staff needed clarity on how CARS and NEWS would work alongside each other. SU/C has mixed views about the extent to the score should be shared with patients.

As far as we are aware previous studies on the design, development and implementation of risk scores have not reported on the views of staff and SU/C, so we are unable to determine the extent implementation of these risk-scoring systems into routine clinical practice requires careful consideration of the views of staff and patients. However, our broad recruitment and data saturation suggests our findings may be generalizable to the implementation of risk scores elsewhere. The themes identified highlight risk scores are complex interventions introduced into complex adaptive systems and the voice of staff and SU/C is an important element of co-design. The contribution of staff and SU/C was integral and iterative to the design of CARS and led to some important insights and design changes including: (1) CARS will update over time and be available as a graph with all its subcomponents also shown. (2) The relationship of CARS with NEWS was important to clarify. We have now designed our risk score to use NEWS in the first instance and then incorporate blood test results as and when available. About ¼ of patients do not have a blood test results. (3) We have decided to present the score as a % (0 to 100) without descriptive labels (e.g. low/medium/high). (4) The score will be visible on the electronic patient record but will not be a "pop-up" alert. We are now working with both NHS trusts, taking a staged approach to implementing CARS as a quality improvement programme and we continue to involve staff and SU/Cs. The qualitative work reported here continues to map the process, identify early problems and support solutions.

For peer review only - http://bmjopen.bmj.com/site/about/guidelines.xhtml

The process of involvement of stakeholders within interventions is challenging and we can usefully reflect on the limitations of our efforts. For staff, finding time for their quality input was hard and whilst we achieved this through flexible approaches to recruitment, venues and timings, the process revealed warnings about the “unfinished business” and “unanswered questions” staff still have about implementation. With respect to SU/Cs, we can refer to notions of an involvement hierarchy of “consultation, collaboration, and user-control” [10] to critique our approach. We predominantly “consulted”, however, we extended this to “collaboration” with our steering group members who maintained input throughout. Much of the implementation and research took place at individual sites, integrated into daily hospital working, mirroring iterative quality improvement process. Including SU/Cs in this is fraught with difficulty and is currently rare in healthcare [11]. Nevertheless as healthcare is increasingly using computer aided decision support systems as a key to achieving gains in quality and patient safety [12], we suggest co-design is necessary to maximise the successful implementation.

Conclusions

Staff and patients had important, often complex, insights to support the development and implementation of CARS which need to be addressed if CARS is to be successfully used in routine practice.

For peer review only - http://bmjopen.bmj.com/site/about/guidelines.xhtml

Contributorship

MAM and DR had the original idea for this work. NJ was the study coordinator. JD, CM and NJ were the leads for the qualitative study. JD, NJ, CM and MAM wrote the first draft of this paper. All authors (including AS and MF) subsequently assisted in redrafting and have approved the final version.

Data Sharing

No additional data available.

Funding statement:

This study is funded by the Health Foundation and the National Institute for Health Research (NIHR) Yorkshire and Humber Patient Safety Translational Research Centre (NIHR Yorkshire and Humber PSTRC). The views expressed in this article are those of the author(s) and not necessarily those of the Health Foundation, the NHS, the NIHR, or the Department of Health and Social Care. The Health Foundation is an independent charity working to improve the quality of health care in the UK.

Competing Interests

No competing interest for any author.

For peer review only

For peer review only - http://bmjopen.bmj.com/site/about/guidelines.xhtml

References

1. Hogan, H., Healey, F., Neale, G., Thomson, R., Vincent, C. and Black, N., 2012. Preventable deaths due to problems in care in English acute hospitals: a retrospective case record review study. *BMJ Qual Saf*, pp.bmjqs-2012.
2. Brabrand, M., Folkestad, L., Clausen, N.G., Knudsen, T. and Hallas, J., 2010. Risk scoring systems for adults admitted to the emergency department: a systematic review. *Scandinavian journal of trauma, resuscitation and emergency medicine*, *18*(1), p.8.
3. Silcock, D.J., Corfield, A.R., Gowens, P.A. and Rooney, K.D., 2015. Validation of the National Early Warning Score in the prehospital setting. *Resuscitation*, *89*, pp.31-35.
4. McGinley, A. and Pearse, R.M., 2012. A national early warning score for acutely ill patients. *British Medical Journal,* 34,; e5310.
5. Faisal, M., Scally, A., Jackson, N., Richardson, D., Beatson, K., Howes, R., Speed, K., Menon, M., Daws, J., Dyson, J., Marsh, C., Mohammed, M.A., Development of a novel computer aided risk score to predict the risk of death in hospital for acutely ill medical patients using their first blood test results and vital signs after admission, *BMJ Open*, in press.
6. Williams, B., Alberti, G., Ball, C., Bell, D., Binks, R. and Durham, L., 2012. National Early Warning Score (NEWS): Standardising the assessment of acute-illness severity in the NHS. *London: The Royal College of Physicians*.
7. Department of Health, 2013, National Early Warning Score, National Clinical Guideline No. 1, https://health.gov.ie/wp-content/uploads/2015/01/NEWSFull-ReportAugust2014.pdf, last accessed July 2018
8. Guest, G., Bunce, A. and Johnson, L., 2006. How many interviews are enough? An experiment with data saturation and variability. *Field methods*, *18*(1), pp.59-82.
9. Braun, V. and Clarke, V., 2006. Using thematic analysis in psychology. *Qualitative research in psychology*, *3*(2), pp.77-101.
10. Hanley B, Bradburn K, Barnes M, Evans C, Goodare H, Kelson M, Kent A, Oliver S, Thomas S & Wallcraft J (2004) Involving the Public in NHS, Public Health and Social Care Research: Brieifing Notes for Researchers, 2nd Edn. INVOLVE. Hampshire.
11. Green SA, Carnegie W, Barber S, Matthew D, Matthews R. Co-designing within quality improvement initiatives: Notes from the field. JHD. 2018;3(1):49–56. https://doi.org/10.21853/JHD.2018.3
12. Baker, A., 2001. Crossing the quality chasm: a new health system for the 21st century. *BMJ: British Medical Journal*, *323*(7322), p.1192.

For peer review only - http://bmjopen.bmj.com/site/about/guidelines.xhtml

Figure 1: Focus group questions round 1

Staff focus group questions	Service User/Carer focus group questions
**General:** What are your thoughts on the CARS? How might the score be valuable? How might the score present challenges? Can you see any problems implementing the score in practice?<br>**Knowledge related to and presentation of the score:** What things do you still need to know about the CARS? What information do you think health care practitioners will need to use the score? How do you think this (information/score) is best disseminated to health care practitioners? How much weight might be attributed to the score in terms of clinical judgement? Talk us through how this might work. How might the score combine with clinical examination and patient reported symptoms?<br>**Components of the score:** Would you share your thoughts how accurate (reliable/valid) you consider the CARS to be based on the information/presentation we have given?<br>**Your responses to CARS:** Talk us through the likely process of action from receiving the CARS. How might the score might aid practitioners' decisions about treatment and care? Can you think of any circumstances where the score might support or undermine your clinical decision? Would you share the CARS score with patients? Relatives and carers? Are there any resource implications associated with the CARS?<br>**Organisational matters:** How might the CARS be used by the institution with regard to resource management? Is there any value to having score recorded in medical records? Any problems?	**General:** What are your thoughts and feelings about the CARS? How might the score be valuable? Do you have any worries about the score?<br>**Awareness:** What information about the score generally do you think people would like? What are your thoughts on patients being told about the score? What about relatives and carers?<br>**Impact on care:** The proposition is that the score might aid practitioners in treatment and care choices (e.g. admit/discharge home, where to admit, active treatment, supportive care)? How does this proposition sound to you? Does this proposition raise any concerns for you? How do you see the patient and carers role in this might be?<br>**Organisational matters:** How might the CARS be impact on resources/resource management or organisation of care?<br>**The following sentences were copied onto cards and presented in turn**<br>In response to the CARS practitioners may. . . . .<br>In response to the CARS I (as a patient)/patients may . . . .<br>In response to the CARS I (as SU/C)/my family . . . . . .<br>The CARS may be valuable for. . . . .<br>The problems with the CARS might be. . . . . .<br>If I knew my/my relative's CARS I might feel . . . .<br>The value for practitioners with regard to the CARS might be. . . . .<br>The problems for practitioners with regard to CARS might be. . . . .<br>Practitioners should share the score with patients/carers when . . . .<br>The best way to communicate the score might be. . . . . . .<br>Putting a number on my risk of deterioration is . . . .

For peer review only

Figure 2: Focus group content round 2

Vignette example

Patient Age (years):	[85]
Gender:	[Male]
time of admission:	[17:25]
Mode of presentation:	[Acute/Emergency]

Description of presentation

Recent previous admission with AKI and CKD, admitted as wife not coping – patient doubly incontinent with very poor mobility and new diarrhoea, past medical history of dementia, T2DM, previous stroke and osteoarthritis, on examination abdominal soft and non-tender, chest clear, heart sounds normal, commenced on IV fluids

Time of Index NEWS:	00:50 NEWS [1]
Respiratory Rate:	[18] breaths per minute
Oxygen Saturations	[97] %
Temperature	[35.1] degrees Celsius
Systolic BP	[150] mmHg
Diastolic BP	[65] mmHg
Pulse Rate	[75] beats per minute
Level of consciousness	[Alert]

Index blood test results

Blood Test	Result
Albumin (g/L)	[35]
Creatinine (umol/L)	[986]
Haemoglobin (g/dl)	[103]
Potassium (mmol/L)	[5.2]
Sodium (mmol/L)	[142]
Urea (mmol/L)	[38.2]
White blood cell count (10^9 cells/L)	[5.5]
Platelets (10^3/microliter)	[281]

Acute Kidney Injury Score [3]

Focus group questions

Response to Vignette: Talk us through your decisions regarding the vignette (use up to 3 vignettes per group).

Response to CARS: The CARS score for this patient is [present score]. What are your thoughts on CARS given this context? Would the score change the decisions you made about treatment and care? How much would you attribute to the score in terms of clinical judgement? How might the score combine with clinical examination and patient reported symptoms?

Knowledge related to the score: How do you think this CARS is best implemented?

Presentation of the score: What did you think to the way we presented the score (scale of 1 to 8 with descriptors such as low, low, moderate and high)? How could it be better presented?

Finally, visual mock-ups of the score were presented and comments requested.

For peer review only

Figure 3: Staff Focus Group Participants

Practitioner	Round 1		Round 2						Total
	A[1]	B[2]	A[3]	A[4]	A[5]	B[6]	B[7]	B[8]	
	n=7	n=10	n=16	n=2	n=3	n=3	n=2	n=2	n=45
Doctor (Dr)	5	2	6	0	3	1	0	0	17
(Senior (Sr) doctor (Dr) consultant/senior registrar)	(3)	(2)	(6)	(0)	(0)	(1)	(0)	(0)	(12)
(Junior doctor (Jr) registrar, FY2, FY1)	(2)	(0)	(0)	(0)	(3)	(0)	(0)	(0)	(5)
Ward based Nurse (N)	0	2	6	2	0	1	2	2	15
Senior (Sr) Nurse (N) (above band 6)	(0)	(2)	(5)	(2)	(0)	(0)	(2)	(0)	(11)
Junior (Jr) Nurse (N) (below band 6)	(0)	(0)	(1)	(0)	(0)	(1)	(0)	(2)	(4)
Nurse Specialist (NS)	2	5	0	0	0	0	0	0	7
Health Care Assistant (HCA)	0	0	2	0	0	0	0	0	2
Other (O) (allied professionals)	0	1	2	0	0	1	0	0	4

NB: A/B is Trust A/B. [] is the count of number of focus groups. n= is the number of participants.

For peer review only

Figure 4: Themes resulting from data analysis according to the study aims

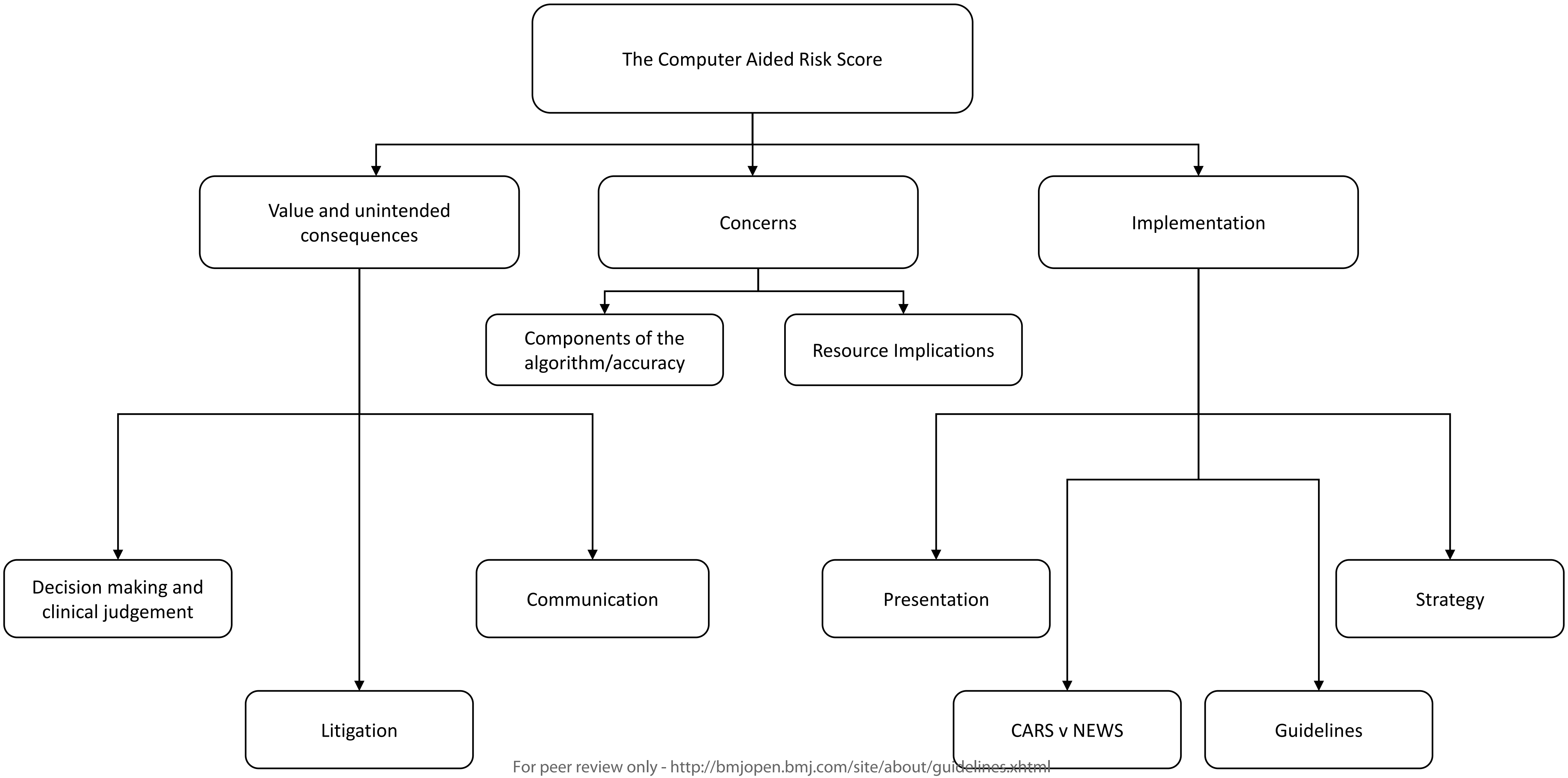

Standards for Reporting Qualitative Research (SRQR)*

http://www.equator-network.org/reporting-guidelines/srqr/

Title and abstract

	Page/line no(s).
**Title** - Concise description of the nature and topic of the study Identifying the study as qualitative or indicating the approach (e.g., ethnography, grounded theory) or data collection methods (e.g., interview, focus group) is recommended	Page 1 lines 1-3<br>Page 3 lines 1-3
**Abstract** - Summary of key elements of the study using the abstract format of the intended publication; typically includes background, purpose, methods, results, and conclusions	Page 1

Introduction

**Problem formulation** - Description and significance of the problem/phenomenon studied; review of relevant theory and empirical work; problem statement	Page 3
**Purpose or research question** - Purpose of the study and specific objectives or questions	Page 3 para 2 and 3

Methods

**Qualitative approach and research paradigm** - Qualitative approach (e.g., ethnography, grounded theory, case study, phenomenology, narrative research) and guiding theory if appropriate; identifying the research paradigm (e.g., postpositivist, constructivist/ interpretivist) is also recommended; rationale**	Page 4 para 5 and page 5 para 1
**Researcher characteristics and reflexivity** - Researchers' characteristics that may influence the research, including personal attributes, qualifications/experience, relationship with participants, assumptions, and/or presuppositions; potential or actual interaction between researchers' characteristics and the research questions, approach, methods, results, and/or transferability	Last para of page 4 and first para of page 5
**Context** - Setting/site and salient contextual factors; rationale**	Page 4 para's 3 and 4 under the heading "participants"
**Sampling strategy** - How and why research participants, documents, or events were selected; criteria for deciding when no further sampling was necessary (e.g., sampling saturation); rationale**	Page 5 paras 3 and 4 under the title "procedure"
**Ethical issues pertaining to human subjects** - Documentation of approval by an appropriate ethics review board and participant consent, or explanation for lack thereof; other confidentiality and data security issues	Page 4 para 1 under the title "ethical approval"
**Data collection methods** - Types of data collected; details of data collection procedures including (as appropriate) start and stop dates of data collection and analysis, iterative process, triangulation of sources/methods, and modification of procedures in response to evolving study findings; rationale**	Page 5 and 6 under the title "analysis"

For peer review only - http://bmjopen.bmj.com/site/about/guidelines.xhtml

**Data collection instruments and technologies** - Description of instruments (e.g., interview guides, questionnaires) and devices (e.g., audio recorders) used for data collection; if/how the instrument(s) changed over the course of the study	Figures 1 and 2 and page 5 and 6 under the title "analysis"
**Units of study** - Number and relevant characteristics of participants, documents, or events included in the study; level of participation (could be reported in results)	Page 6 under the title "characteristics of the sample" and figure 3
**Data processing** - Methods for processing data prior to and during analysis, including transcription, data entry, data management and security, verification of data integrity, data coding, and anonymization/de-identification of excerpts	Page 5 and 6 under the title "analysis"
**Data analysis** - Process by which inferences, themes, etc., were identified and developed, including the researchers involved in data analysis; usually references a specific paradigm or approach; rationale**	Page 5 and 6 under the title "analysis"
**Techniques to enhance trustworthiness** - Techniques to enhance trustworthiness and credibility of data analysis (e.g., member checking, audit trail, triangulation); rationale**	

Results/findings

**Synthesis and interpretation** - Main findings (e.g., interpretations, inferences, and themes); might include development of a theory or model, or integration with prior research or theory	Page 6 lines 1-2
**Links to empirical data** - Evidence (e.g., quotes, field notes, text excerpts, photographs) to substantiate analytic findings	Page 7 to 12

Discussion

**Integration with prior work, implications, transferability, and contribution(s) to the field -** Short summary of main findings; explanation of how findings and conclusions connect to, support, elaborate on, or challenge conclusions of earlier scholarship; discussion of scope of application/generalizability; identification of unique contribution(s) to scholarship in a discipline or field	Page 12 to 14
**Limitations** - Trustworthiness and limitations of findings	

Other

**Conflicts of interest** - Potential sources of influence or perceived influence on study conduct and conclusions; how these were managed	Page 13 para 3 and page 14 para 1
**Funding** - Sources of funding and other support; role of funders in data collection, interpretation, and reporting	Page 15 under the title "acknowledgements"

*The authors created the SRQR by searching the literature to identify guidelines, reporting standards, and critical appraisal criteria for qualitative research; reviewing the reference lists of retrieved sources; and contacting experts to gain feedback. The SRQR aims to improve the transparency of all aspects of qualitative research by providing clear standards for reporting qualitative research.

For peer review only

**The rationale should briefly discuss the justification for choosing that theory, approach, method, or technique rather than other options available, the assumptions and limitations implicit in those choices, and how those choices influence study conclusions and transferability. As appropriate, the rationale for several items might be discussed together.

Reference:

O'Brien BC, Harris IB, Beckman TJ, Reed DA, Cook DA. **Standards for reporting qualitative research: a synthesis of recommendations.** *Academic Medicine*, Vol. 89, No. 9 / Sept 2014 DOI: 10.1097/ACM.0000000000000388

For peer review only

For peer review only - http://bmjopen.bmj.com/site/about/guidelines.xhtml

BMJ Open

Understanding and applying practitioner and patient views on the implementation of a novel automated Computer Aided Risk Score (CARS) predicting the risk of death following emergency medical admission to hospital: A qualitative study

Journal:	*BMJ Open*
Manuscript ID	bmjopen-2018-026591.R2
Article Type:	Research
Date Submitted by the Author:	07-Mar-2019
Complete List of Authors:	Dyson, Judith; University of Hull, Health and Social Work<br>Marsh, Claire; Bradord Institute for Health Research, Quality and Safety<br>Jackson , Natalie ; Bradord Institute for Health Research, Quality and Safety<br>Richardson, Donald; York Teaching Hospital NHS Foundation Trust Hospital, Renal Medicine<br>Faisal, Muhammad; University of Bradford, Faculty of Health Studies; University of Bradford<br>Scally, Andrew ; University of Bradford, School of Health Studies<br>Mohammed, Mohammed ; University of Bradford, School of Health Studies
**Primary Subject Heading**:	Qualitative research
Secondary Subject Heading:	Evidence based practice, Health services research
Keywords:	Computer Aided Risk Score, Implementation, Service User Involvement, CARS, Early warning score, Hospital Mortality

SCHOLARONE™
Manuscripts

Understanding and applying practitioner and patient views on the implementation of a novel automated Computer Aided Risk Score (CARS) predicting the risk of death following emergency medical admission to hospital: A qualitative study

Corresponding Author: Dr Judith Dyson, University of Hull, Allamm Medical Building Room 331, Cottingham Road, HULL, UK HU6 7RX, T: 01482 463806, E: j.dyson@hull.ac.uk

Dr Claire Marsh, Bradford Institute of Health Research, Bradford UK
Ms Natalie Jackson, Bradford Institute of Health Research, Bradford UK
Dr Donald Richardson, York Teaching Hospitals NHS Foundation Trust, York UK
Dr Muhammad Faisal, University of Bradford, Bradford, UK
Dr Andrew Scally, University of Bradford, Bradford, UK
Prof Mohammed A Mohammed, University of Bradford, Bradford, UK

Word count 3997

For peer review only - http://bmjopen.bmj.com/site/about/guidelines.xhtml

Understanding and applying practitioner and patient views on the implementation of a novel automated Computer Aided Risk Score (CARS) predicting the risk of death following emergency medical admission to hospital: A qualitative study

Abstract (281 words)

Objectives: The Computer Aided Risk Score (CARS) estimates the risk of death following emergency admission to medical wards using routinely collected vital signs and blood test data. Our aim was to elicit the views of health care practitioners (staff) and Service Users and Carers (SU/C) on i) the potential value, unintended consequences and concerns associated with CARS and practitioner views on ii) the issues to consider before embedding CARS into routine practice.

Setting: This study was conducted in two NHS Hospital Trusts in the North of England. Both had in-house IT development teams, mature IT infrastructure with electronic National Early Warning Score (NEWS) and were capable of integrating NEWS with blood test results. The study focused on emergency medical and elderly admissions units. There were 60 and 39 acute medical/elderly admissions beds at the two NHS hospital Trusts.

Participants: We conducted eight focus groups with 45 health care practitioners and two with 11 service users and carers (SU/Cs) in two NHS acute hospitals.

Results: Staff and SU/Cs recognised the potential of CARS but were clear the score should not replace or undermine clinical judgments. Staff recognised CARS could enhance clinical decision making/judgments and aid communication with patients. They wanted to understand the components of CARS and be reassured about its accuracy but were concerned about the impact on intensive care and blood tests.

Conclusion: Risk scores are widely used in healthcare, but their development and implementation does not usually involve input from practitioners and SU/Cs. We contributed to the development of CARS by eliciting views of staff and SU/Cs who provided important, often complex, insights to support the development and implementation of CARS to ensure successful implementation in routine clinical practice.

For peer review only - http://bmjopen.bmj.com/site/about/guidelines.xhtml

Strengths and limitations

- Our research takes a rare approach of including healthcare practitioner and service user/carer (SU/C) involvement in the development of a risk score
- Finding adequate time for practitioner input was hard and we needed flexible approaches to focus group recruitment, venues and timings
- Staff and SU/C input was largely a process of our consulting with the group. A co-design approach may have enhanced the benefits of stakeholder involvement.

For peer review only

For peer review only - http://bmjopen.bmj.com/site/about/guidelines.xhtml

Understanding and applying practitioner and patient views on the implementation of a novel automated Computer Aided Risk Score (CARS) predicting the risk of death following emergency medical admission to hospital: A qualitative study

Dr Judith Dyson, University of Hull, Cottingham Road, HULL, UK HU6 7RX, T: 01482 463806, E: j.dyson@hull.ac.uk
Dr Claire Marsh, Bradford Institute of Health Research, Bradford UK
Ms Natalie Jackson, Bradford Institute of Health Research, Bradford UK
Dr Donald Richardson, York Teaching Hospitals NHS Foundation Trust, York UK
Dr Muhammad Faisal, University of Bradford, Bradford, UK
Dr Andrew Scally, University of Bradford, Bradford, UK
Prof Mohammed A Mohammed, University of Bradford, Bradford, UK

Key Terms: "Computer Aided Risk Score", CARS, "Service User Involvement", Implementation, "Early Warning Score", "Hospital Mortality"

Word count: 3997

Introduction

A UK wide study of 10 hospitals estimated 5% of deaths were preventable and 30% of these were attributable to poor clinical monitoring [1]. If risk of death information was available to clinical staff it was likely to enhance patient safety [2], however, there are no established risk equations for acutely admitted medical patients. Furthermore, while several studies have considered the use of physiological signs *or* blood tests in the assessment of patient few consider combining the two [2]. Although NEWS is known to predict mortality in the hospital and pre-hospital setting [3] it is not suitable for some groups of patients [4]. This research team therefore developed a novel computer aided risk score (CARS) for estimating the risk of in-hospital mortality following emergency medical admission to hospital [5] in two hospitals. CARS was designed to rely on variables already routinely collected and electronically recorded as part of the process of care including vital signs data (based on a National Early Warning Score (NEWS))[6] and blood test results [7]. CARS demonstrated better discrimination and calibration than blood tests and NEWS separately [5].

Despite the widespread use of risk scores to enhance decision making in healthcare, as identified by Braband et al [2], there is little or no documentary evidence of the involvement of healthcare practitioners and service users or carers (SU/Cs) in the design, development and implementation of this type of risk score. Our research responds to this gap.

For peer review only - http://bmjopen.bmj.com/site/about/guidelines.xhtml

For peer review only

Concurrent to the statistical modelling work, we conducted focus groups with healthcare practitioners and SU/Cs to feed into the on-going development of CARS. The aims of the focus groups were to establish i) health care practitioner (hereafter "staff") and SU/C views on the potential value, unintended consequences and concerns associated with the development of the CARS and ii) staff views on how CARS should be adopted in practice/implementation needs

Methods

Ethical approval

Ethical approval was granted from National Research Ethics Service (15/YH/0348) and Research Governance approval from the trusts involved in the study. All participants gave signed consent after receiving written information about the study.

Patient and Public Involvement

The "Service User and Carer Involvement in Research Group" (SURG) at the University of Bradford supported the project as members of the project steering group and as a focus group advisory team. Their contribution included co-design of project materials, support of the methodology (e.g. recruitment strategies) and offering comments and suggestions based on data gathered.

Participants

This study was conducted in two NHS Hospital Trusts in the North of England (referred to hereafter as Trust A and Trust B). Both had in-house IT development teams, mature IT infrastructure with electronic NEWS and were capable of integrating NEWS with blood test results. The study focused on emergency medical and elderly admissions units. There were 60 and 39 acute medical/elderly admissions beds at Trusts A and B respectively.

SU/Cs were competent adults (aged over 18 years) who were members of the public, who had either been in hospital themselves any time in their adult life, or who had experienced a relative in hospital. Staff were, any practitioner working in areas where we intended to implement CARS (acute assessment units, medical wards and older person in-patient units) or acute outreach staff (nurse or doctor called upon to offer advanced assessment and input), were eligible. Due to the additional aim of the staff groups (implementation needs) we held separate SU/C and staff focus groups.

For peer review only - http://bmjopen.bmj.com/site/about/guidelines.xhtml

Design

Ten focus groups were held over two rounds in each Trust (eight staff groups and two SU/C groups). A first round of staff and SU/C focus groups was conducted at the beginning of CARS development and commenced with a brief presentation about CARS, its rationale and development, then asked participants for their thoughts, feelings and concerns in relation to implementation of a CARS at their hospitals. Focus group schedules were informed by the literature relating to other risk scores (e.g. NEWS). Results from focus groups were fed back to the CARS research team who then further developed CARS and its implementation package (Figure 1 illustrates round one focus group questions). Subsequently, a second round of focus groups with staff explored CARS implementation needs in greater depth. After a presentation about CARS, vignettes were used (developed from case note reviews), to allow staff to understand how CARS scores relate to real clinical scenarios (Figure 2 offers an example of a vignette and round two focus group questions). It was our intention to run groups of between 6 and 12 participants. Due to the challenges of staff time and availability, this was revised to smaller group sizes for the second staff focus groups. JD led staff focus groups, CM led SU/C groups and NJ supported all groups (none of whom had a previous relationship with participants and all were experienced in running focus groups).

Figure 1: Focus group questions round 1

Figure 2: Focus group content round 2

Procedure

CM approached the Patient Experience leads at each hospital as a gatekeeper for recruitment to SU/Cs. Patient Experience leads contacted members of their forums through email, posters and verbal invites. Interested people contacted CM directly and were given participant information sheets before deciding to attend. Clinical partners from the CARS implementation teams at both hospitals introduced JD and NJ (electronically or in person) to the nurse in charge of relevant hospital areas. Charge nurses circulated email invitations to qualified medical and nursing staff of all grades with participant information. They contacted the research team if they were interested in participating. All interested SU/C and staff were included.

The first round of focus groups (SU/C and staff) took place over a six-month period from May 2016 and the second staff focus groups occurred between May and July 2017. For staff, careful recruitment identified a diverse range of participants in terms of their professional role and experience. There were slightly more medical staff, which was expected given the relevance of the score. We expected

For peer review only - http://bmjopen.bmj.com/site/about/guidelines.xhtml

focus groups including 25 participants within groups would be enough to achieve data saturation [8]. With one exception, all focus groups took place in hospital meeting rooms or offices. The exception was one staff focus group, which took place at a conference centre (for the convenience of staff).

Analysis

Focus groups were audio recorded and transcribed verbatim and transcripts imported into NVivo v.11 data management software. Data were subject to thematic analysis [9]. An inductive approach generated themes. Data were coded by one main coder (JD) but to increase reliability of analysis SU/C focus groups data were also coded by CM and a sample of staff focus group data coded by NJ. Coding was sentence by sentence to allow accurate comparison. Differences in coding were discussed and where necessary codes were re-defined and the process repeated. On the second occasion over 90% agreement was reached on codes allocated. Coding was according to the three areas of interest, value and unintended consequences, concerns and implementation. Data saturation was achieved; no new codes were derived from data from the last two focus groups.

Results

Characteristics of the sample

SU/C groups in NHS trusts A and B involved six and five participants respectively. The composition of the staff groups was according to figure 3; junior doctor refers to doctors in their first (FY1) or second (FY2) year post qualification or registrar (first promotion post qualifying). Senior doctor refers to senior registrar (pre-consultant grade) and consultant (most senior medical person). Grades of nurses include below six (five being the most junior post qualification nurse) and above six (seven charge nurse, eight matron and above nurse specialists or clinical managers). Allied professionals included physio and occupational therapists. We did not formally ask SU/Cs about the nature of their or their friend/relative's hospital stay, however, examples given to support views offered suggests a wide range of experiences (e.g. heart bypass surgery, caring at the end of life, chest infection and subsequent pneumonia). SU/Cs talked about admissions to the Emergency Department, Intensive Care Unit and both medical and surgical wards. There were eight staff focus groups with the number of participants ranging from two to 16 across both Trusts. There were six and five participants in SU/C groups in trusts A and B respectively. All participants contributed to focus groups. The duration of focus groups ranged between 22 minutes and one hour 29 minutes, mean duration 57 minutes.

Figure 3: Staff Focus Group Participants

Overall Findings

For peer review only - http://bmjopen.bmj.com/site/about/guidelines.xhtml

There were nine themes arranged according to the aims of the study, "value and unintended consequences", "concerns" and "implementation" represented in figure 4 and elaborated below with verbatim quotes from a broad range of participants.

Figure 4: Themes resulting from data analysis according to the study aims

Value and unintended consequences

Decision-making and clinical judgement

Staff talked about the value of using CARS as a decision aid for choosing active or supportive care or for "do not resuscitate" decisions.

> *" . . . on admission . . .might help triage"* Sr Dr1 FG1

> *". . . decisions about end of life care as well; guide DNR* [do not resuscitate] *decision making"* Sr Dr3, FG1

This was considered within different *contexts* of care; in some areas a high score might suggest supportive care (e.g. general medical areas) and in other areas (e.g. paediatrics) it would be expected even the smallest chance of survival suggests active care.

> *"some clinicians would say even half a percent chance of survival is enough. . . . where do we draw the line?"* Sr Dr3, FG1

Staff considered the score would give them extra confidence in making clinical decisions.

> *"Having the CARS then makes you think no ok I am on the right way and it gives you a bit more confidence"* Sr N1, FG3

> *"I think CARS score, well all scoring systems are useful anyway as they are a starting point to see how sick your patient is, it's a good way of just getting all the information in one go"* Sr Dr1, FG3

SU/C groups were similarly positive about its potential to play a role in improving patient safety because it could challenge clinician's preconceived ideas and provide additional information for clinicians to make a judgement.

> *"what I've seen is that they get this idea in their head of what's wrong with you and this is perhaps a good way of making double check"* SU/C Trust B

Both staff and SU/Cs expressed concerns clinical judgement may be undermined by the score. Staff discussions focused on the appropriate "weight" to give the score in the decision making process particularly when the score and their own judgement conflicted.

For peer review only - http://bmjopen.bmj.com/site/about/guidelines.xhtml

"We want the space to use that judgement . . ." Jr Dr1 FG1

"With all of these things [scores] *you stop taking a clinical interest in the patient and just look at the numbers"* NS1 FG2

"as long as it's another helpful factor in deciding what to do as opposed to being the determining factor" SU/C Trust A

Litigation

Staff saw potential positive and negative elements to the score in terms of supporting their decisions or otherwise and the potential for litigation.

"If you need to back up your clinical judgement to the coroner [the Computer Aided Risk Score] *would help I think"* Sr Dr2 FG1

"Someone is going to say, I am going to pore over those notes and find out why this person has died, whereas, they may not have done previously. Conversely this person had a high chance of dying, why did you carry on with your treatment which . . . was futile" Sr Dr1 FG1

Communication

Most staff considered the score would aid or prompt communication with patients about prognosis. There was disagreement about it might be best to give the actual score or a description of the situation.

"We tend to use more descriptive terms . . . patients are appreciative of honesty" Sr Dr1 FG1

"If he had a score, today this is how bad she actually is its likely going to be soon, that would have helped him deal with the situation better" SU/C Trust A

SU/C participants talked at length about the score's potential role in assisting communication about a patient's condition, helping them accept the seriousness of the condition. However, there was much debate amongst both staff and SU/Cs about whether or not patients and carers should be actively informed of the score.

"One of the biggest things in any hospital anywhere is communication and information they're not told, and I'm sure they would like to be told" SU/C Trust B

"They [staff] *are a little too tactful, little too polite, little too sensitive . . the score might help"* SU/C Trust B

It was argued this score was primarily for clinicians and whilst it should not hidden, it need not be routinely provided in the same format for everyone.

For peer review only - http://bmjopen.bmj.com/site/about/guidelines.xhtml

> *"I think if the family are told they are gravely ill that would be more human than they are an eight point four"* SR Dr1 FG2

Components of the algorithm/accuracy

Staff discussed at length the need to know the component parts of the score and access the latest contributing values. They were keen to know when CARS may or may not be accurate. Frequent examples offered of where CARS may not be accurate were Chronic Obstructive Pulmonary Disease (COPD), Chronic Kidney Disease (CKD) terminal conditions and congenital diseases.

> *"I would like the people who are reviewing the score to be able to understand it properly. Otherwise you will get people who are becoming overly worried about it when they don't actually, can't interpret it and don't understand it."* Jr Dr1 FG5

> *"What about COPD* [Chronic Obstructive Pulmonary Disease] *. . . at the same time people with significant co-morbidities will have a worse outcome?"* Jr Dr2 FG1

SU/C groups were also interested in exploring the accuracy of the score:

> *"It needs to be sensitive both ways . . . otherwise everybody will be in the high dependency unit"* SU/C Trust B

Resource Implications

Staff raised questions about the potential resource needs associated with CARS e.g. intensive Care Unit beds and blood tests.

> *"Can I just ask what sort of impact this will have on the labs?"* NS2 FG2

> *"the extra expenditure. . . you have an ethical dilemma because you have a patient who's got a score you've gone to escalating to HDU* [High Dependency Unit], *ICU* [Intensive Care Unit], *high observations units."* NS3 FG2

SU/C groups were also concerned about resources but this focused on extra workload and they were worried the score would mean less face-to-face care.

> *"My concerns would be they're already under extreme pressure, if this is going to be another assessment that they have got to carry out on patients that's increasing the pressure at a time when they're already stressed out"* SU/C Trust B

Implementation

Presentation

Staff focus groups had ideas as to the presentation of CARS and the need to see the trend in the score.

> *"If I were using it myself as a physician I would want a specific percentage"* Jr Dr1 FG1

For peer review only - http://bmjopen.bmj.com/site/about/guidelines.xhtml

"Putting it all together in a score is helpful when you are the person on call who doesn't know them and you can see the trend of that score and it's helpful." Jr Dr1 FG5

"It would be useful to see it as a graph [trend]" Jr Ns1 FG8

When discussing presentation of the score the SU/Cs focused mainly on the communication of it by staff (reported above) and whether they should have direct access/sight of the score.

"To see it change in front of your eyes that might be even more terrifying" SU/C Trust A

CARS compared with NEWS

Staff appreciated CARS was more sensitive than NEWS, and though they appreciated, unlike NEWS, the CARS was a complex statistical equation, not possible to calculate by hand, they were keen to see it.

"Can you copy that algorithm? Can we have a look at that?" NS3 FG2 Trust B

The key issue with respect to this comparison was the potential for CARS to suggest one action should be taken and NEWS suggests another (e.g. one indicates escalation the other does not).

"NEWS [National Early Warning Score] *score high and CARS low or vice versa therefore we've then got a confusion to the people who are actually on the shop floor where one thing is telling them to do this escalation and the other is saying you don't need to"* O FG2

Staff considered the comparative utility of NEWS and CARS with a particular focus on whether blood tests would delay a calculation of the score, or, whether CARS would be updated when any new data item (e.g. temperature/pulse) became available.

"[CARS unlike NEWS] *it might take three or four hours .. .if it relies on blood tests"* Sr Dr3 FG1

Finally, there was indication staff wanted to see the score demonstrated to be effective in relation to *people* in addition to being mathematically valid.

Guidelines

Staff discussed the specific procedures for CARS' role in confirming or support clinical judgement:

"I don't think you can use the CARS score as a trigger to make any specific clinical action, it is an alert that there may be a clinical problem there, there is a clinical problem there and then you need to find out what it actually is, it may be you need to look more closely at the biochemistry or whatever, whereas, the NEWS score is more specific" NS4 FG2

For peer review only - http://bmjopen.bmj.com/site/about/guidelines.xhtml

They initially suggested the need for an escalation protocol or guide (where actions are prescribed according to score).

> *". . . with the NEWS* [National Early Warning Score] *if you have a score of five an above obviously that is an escalation process whereas with the CARS we don't know"* NS1 FG2

When vignettes were brought in during the second staff focus groups, staff were less likely to feel the need for an escalation protocol or a guide.

> *"I don't think it* [CARS] *changes the clinical management because the clinical management is always going to be based on the individual in front of you with their individual bloods and things. Putting it all together in a score is helpful"* Jr Dr2 FG5

Where staff wanted guidance this was sometimes to protect against *criticism* about inappropriate response to a high CARS.

> *"If there isn't* [a protocol], *you go to the doctor and say the CARS has come up at this score, and they say yes that's because. . . . . they would always have a rationale . . . but at least you are covering yourself"* Sr Ns1 FG7

This was linked to concerns about litigation.

> *"Then one day someone will turn around and say but the CARS score was 10 and you didn't do this, so I think that's just the world we live in and we have to have a clear role when we introduce it or otherwise. . . ."* Jr1 Dr FG 5

Some felt the guidance would ensure (insist upon) a response from a senior clinician.

> *"You can say to the doctor look I am just following the protocol"* Sr Ns1 FG4

Others suggested it would serve as support for more junior staff.

> *"Junior medical and nursing staff . . . I would worry that they would, that they might lack clinical prowess"* Sr Dr3 FG3

Strategy

There was a lot of discussion about a strategy for implementation. One group (FG2) spent a lot of time discussing the need for a "champion". This related to their experience with NEWS. The suggestion was one of the outreach team would be best placed for this. Discussion took place about the extent of education required and there was a consensus a hospital wide strategy would be appropriate.

> *"We need people to champion this"* O FG2

For peer review only - http://bmjopen.bmj.com/site/about/guidelines.xhtml

"The big nooks and crooks is going to be education, needs training, information as well. If you're just looking at one area, there are medical people coming through that area teams to you need to target them all." Sr Dr1 FG2

Discussion

Our approach in developing CARS has been co-working with front line staff and SU/Cs as part of the project team as well as participants. We conducted 10 focus groups with 11 SU/Cs and 45 health care practitioners in two NHS acute hospitals. Participants were interested in the development of the CARS score and appreciated such efforts to improve patient safety at their hospital. They recognised the potential of CARS but were clear the score should not replace or undermine clinical judgments. Staff recognised CARS could enhance clinical decision-making and aid communication. They wanted to understand the components of CARS and be reassured about its accuracy and were concerned about the impact on resources. Staff preferred CARS to be shown as a score (without descriptive labels) graphed by time to monitor changes. Staff needed clarity on how CARS and NEWS would work alongside each other. SU/C has mixed views about the extent to the score should be shared with patients.

As far as we are aware previous studies on the design, development and implementation of risk scores have not reported on the views of staff and SU/C, so we are unable to determine the extent implementation of these risk-scoring systems into routine clinical practice requires careful consideration of the views of staff and patients. However, our broad recruitment and data saturation suggests our findings may be generalizable to the implementation of risk scores elsewhere. The themes identified highlight risk scores are complex interventions introduced into complex adaptive systems and the voice of staff and SU/C is an important element of co-design. The contribution of staff and SU/C was integral and iterative to the design of CARS and led to some important insights and design changes including: (1) CARS will update over time and be available as a graph with all its subcomponents also shown. (2) The relationship of CARS with NEWS was important to clarify. We have now designed our risk score to use NEWS in the first instance and then incorporate blood test results as and when available. About ¼ of patients do not have a blood test results. (3) We have decided to present the score as a % (0 to 100) without descriptive labels (e.g. low/medium/high). (4) The score will be visible on the electronic patient record but will not be a "pop-up" alert. We are now working with both NHS trusts, taking a staged approach to implementing CARS as a quality improvement programme and we continue to involve staff and SU/Cs. The qualitative work reported here continues to map the process, identify early problems and support solutions.

For peer review only - http://bmjopen.bmj.com/site/about/guidelines.xhtml

The process of involvement of stakeholders within interventions is challenging and we can usefully reflect on the limitations of our efforts. For staff, finding time for their quality input was hard and whilst we achieved this through flexible approaches to recruitment, venues and timings, the process revealed warnings about the "unfinished business" and "unanswered questions" staff still have about implementation. With respect to SU/Cs, we can refer to notions of an involvement hierarchy of "consultation, collaboration, and user-control" [10] to critique our approach. We predominantly "consulted", however, we extended this to "collaboration" with our steering group members who maintained input throughout. Much of the implementation and research took place at individual sites, integrated into daily hospital working, mirroring iterative quality improvement process. Including SU/Cs in this is fraught with difficulty and is currently rare in healthcare [11]. Nevertheless as healthcare is increasingly using computer aided decision support systems as a key to achieving gains in quality and patient safety [12], we suggest co-design is necessary to maximise the successful implementation.

Conclusions

Staff and patients had important, often complex, insights to support the development and implementation of CARS which need to be addressed if CARS is to be successfully used in routine practice.

For peer review only

For peer review only - http://bmjopen.bmj.com/site/about/guidelines.xhtml

Contributorship

MAM and DR had the original idea for this work. NJ was the study coordinator. JD, CM and NJ were the leads for the qualitative study. JD, NJ, CM and MAM wrote the first draft of this paper. All authors (including AS and MF) subsequently assisted in redrafting and have approved the final version.

Data Sharing

No additional data available.

Funding statement:

This study is funded by the Health Foundation and the National Institute for Health Research (NIHR) Yorkshire and Humber Patient Safety Translational Research Centre (NIHR Yorkshire and Humber PSTRC). The views expressed in this article are those of the author(s) and not necessarily those of the Health Foundation, the NHS, the NIHR, or the Department of Health and Social Care. The Health Foundation is an independent charity working to improve the quality of health care in the UK.

Competing Interests

No competing interest for any author.

For peer review only

For peer review only - http://bmjopen.bmj.com/site/about/guidelines.xhtml

References

1. Hogan, H., Healey, F., Neale, G., Thomson, R., Vincent, C. and Black, N., 2012. Preventable deaths due to problems in care in English acute hospitals: a retrospective case record review study. *BMJ Qual Saf*, pp.bmjqs-2012.
2. Brabrand, M., Folkestad, L., Clausen, N.G., Knudsen, T. and Hallas, J., 2010. Risk scoring systems for adults admitted to the emergency department: a systematic review. *Scandinavian journal of trauma, resuscitation and emergency medicine*, *18*(1), p.8.
3. Silcock, D.J., Corfield, A.R., Gowens, P.A. and Rooney, K.D., 2015. Validation of the National Early Warning Score in the prehospital setting. *Resuscitation*, *89*, pp.31-35.
4. McGinley, A. and Pearse, R.M., 2012. A national early warning score for acutely ill patients. *British Medical Journal,* 34,; e5310.
5. Faisal, M., Scally, A., Jackson, N., Richardson, D., Beatson, K., Howes, R., Speed, K., Menon, M., Daws, J., Dyson, J., Marsh, C., Mohammed, M.A., Development of a novel computer aided risk score to predict the risk of death in hospital for acutely ill medical patients using their first blood test results and vital signs after admission, *BMJ Open*, in press.
6. Williams, B., Alberti, G., Ball, C., Bell, D., Binks, R. and Durham, L., 2012. National Early Warning Score (NEWS): Standardising the assessment of acute-illness severity in the NHS. *London: The Royal College of Physicians*.
7. Department of Health, 2013, National Early Warning Score, National Clinical Guideline No. 1, https://health.gov.ie/wp-content/uploads/2015/01/NEWSFull-ReportAugust2014.pdf, last accessed July 2018
8. Guest, G., Bunce, A. and Johnson, L., 2006. How many interviews are enough? An experiment with data saturation and variability. *Field methods*, *18*(1), pp.59-82.
9. Braun, V. and Clarke, V., 2006. Using thematic analysis in psychology. *Qualitative research in psychology*, *3*(2), pp.77-101.
10. Hanley B, Bradburn K, Barnes M, Evans C, Goodare H, Kelson M, Kent A, Oliver S, Thomas S & Wallcraft J (2004) Involving the Public in NHS, Public Health and Social Care Research: Brieifing Notes for Researchers, 2nd Edn. INVOLVE. Hampshire.
11. Green SA, Carnegie W, Barber S, Matthew D, Matthews R. Co-designing within quality improvement initiatives: Notes from the field. JHD. 2018;3(1):49–56. https://doi.org/10.21853/JHD.2018.3
12. Baker, A., 2001. Crossing the quality chasm: a new health system for the 21st century. *BMJ: British Medical Journal*, *323*(7322), p.1192.

For peer review only

For peer review only - http://bmjopen.bmj.com/site/about/guidelines.xhtml

Figure 1: Focus group questions round 1

Staff focus group questions	Service User/Carer focus group questions
**General:** What are your thoughts on the CARS? How might the score be valuable? How might the score present challenges? Can you see any problems implementing the score in practice?<br>**Knowledge related to and presentation of the score:** What things do you still need to know about the CARS? What information do you think health care practitioners will need to use the score? How do you think this (information/score) is best disseminated to health care practitioners? How much weight might be attributed to the score in terms of clinical judgement? Talk us through how this might work. How might the score combine with clinical examination and patient reported symptoms?<br>**Components of the score:** Would you share your thoughts how accurate (reliable/valid) you consider the CARS to be based on the information/presentation we have given?<br>**Your responses to CARS:** Talk us through the likely process of action from receiving the CARS. How might the score might aid practitioners' decisions about treatment and care? Can you think of any circumstances where the score might support or undermine your clinical decision? Would you share the CARS score with patients? Relatives and carers? Are there any resource implications associated with the CARS?<br>**Organisational matters:** How might the CARS be used by the institution with regard to resource management? Is there any value to having score recorded in medical records? Any problems?	**General:** What are your thoughts and feelings about the CARS? How might the score be valuable? Do you have any worries about the score?<br>**Awareness:** What information about the score generally do you think people would like? What are your thoughts on patients being told about the score? What about relatives and carers?<br>**Impact on care:** The proposition is that the score might aid practitioners in treatment and care choices (e.g. admit/discharge home, where to admit, active treatment, supportive care)? How does this proposition sound to you? Does this proposition raise any concerns for you? How do you see the patient and carers role in this might be?<br>**Organisational matters:** How might the CARS be impact on resources/resource management or organisation of care?<br>**The following sentences were copied onto cards and presented in turn**<br>In response to the CARS practitioners may. . . . .<br>In response to the CARS I (as a patient)/patients may . . . .<br>In response to the CARS I (as SU/C)/my family . . . . . .<br>The CARS may be valuable for. . . . .<br>The problems with the CARS might be. . . . . .<br>If I knew my/my relative's CARS I might feel . . . .<br>The value for practitioners with regard to the CARS might be. . . . .<br>The problems for practitioners with regard to CARS might be. . . . .<br>Practitioners should share the score with patients/carers when . . . .<br>The best way to communicate the score might be. . . . . . .<br>Putting a number on my risk of deterioration is . . . .

For peer review only

Figure 2: Focus group content round 2

Vignette example

Patient Age (years):	[85]
Gender:	[Male]
time of admission:	[17:25]
Mode of presentation:	[Acute/Emergency]

Description of presentation

Recent previous admission with AKI and CKD, admitted as wife not coping – patient doubly incontinent with very poor mobility and new diarrhoea, past medical history of dementia, T2DM, previous stroke and osteoarthritis, on examination abdominal soft and non-tender, chest clear, heart sounds normal, commenced on IV fluids

Time of Index NEWS:	00:50 NEWS [1]
Respiratory Rate:	[18] breaths per minute
Oxygen Saturations	[97] %
Temperature	[35.1] degrees Celsius
Systolic BP	[150] mmHg
Diastolic BP	[65] mmHg
Pulse Rate	[75] beats per minute
Level of consciousness	[Alert]

Index blood test results

Blood Test	Result
Albumin (g/L)	[35]
Creatinine (umol/L)	[986]
Haemoglobin (g/dl)	[103]
Potassium (mmol/L)	[5.2]
Sodium (mmol/L)	[142]
Urea (mmol/L)	[38.2]
White blood cell count (10^9 cells/L)	[5.5]
Platelets (10^3/microliter)	[281]

Acute Kidney Injury Score [3]

Focus group questions

Response to Vignette: Talk us through your decisions regarding the vignette (use up to 3 vignettes per group).

Response to CARS: The CARS score for this patient is [present score]. What are your thoughts on CARS given this context? Would the score change the decisions you made about treatment and care? How much would you attribute to the score in terms of clinical judgement? How might the score combine with clinical examination and patient reported symptoms?

Knowledge related to the score: How do you think this CARS is best implemented?

Presentation of the score: What did you think to the way we presented the score (scale of 1 to 8 with descriptors such as low, low, moderate and high)? How could it be better presented?

Finally, visual mock-ups of the score were presented and comments requested.

For peer review only

Figure 3: Staff Focus Group Participants

Practitioner	Round 1		Round 2						Total
	A[1]	B[2]	A[3]	A[4]	A[5]	B[6]	B[7]	B[8]	
	n=7	n=10	n=16	n=2	n=3	n=3	n=2	n=2	n=45
Doctor (Dr)	5	2	6	0	3	1	0	0	17
(Senior (Sr) doctor (Dr) consultant/senior registrar)	(3)	(2)	(6)	(0)	(0)	(1)	(0)	(0)	(12)
(Junior doctor (Jr) registrar, FY2, FY1)	(2)	(0)	(0)	(0)	(3)	(0)	(0)	(0)	(5)
Ward based Nurse (N)	0	2	6	2	0	1	2	2	15
Senior (Sr) Nurse (N) (above band 6)	(0)	(2)	(5)	(2)	(0)	(0)	(2)	(0)	(11)
Junior (Jr) Nurse (N) (below band 6)	(0)	(0)	(1)	(0)	(0)	(1)	(0)	(2)	(4)
Nurse Specialist (NS)	2	5	0	0	0	0	0	0	7
Health Care Assistant (HCA)	0	0	2	0	0	0	0	0	2
Other (O) (allied professionals)	0	1	2	0	0	1	0	0	4

NB: A/B is Trust A/B. [] is the count of number of focus groups. n= is the number of participants.

For peer review only

Figure 4: Themes resulting from data analysis according to the study aims

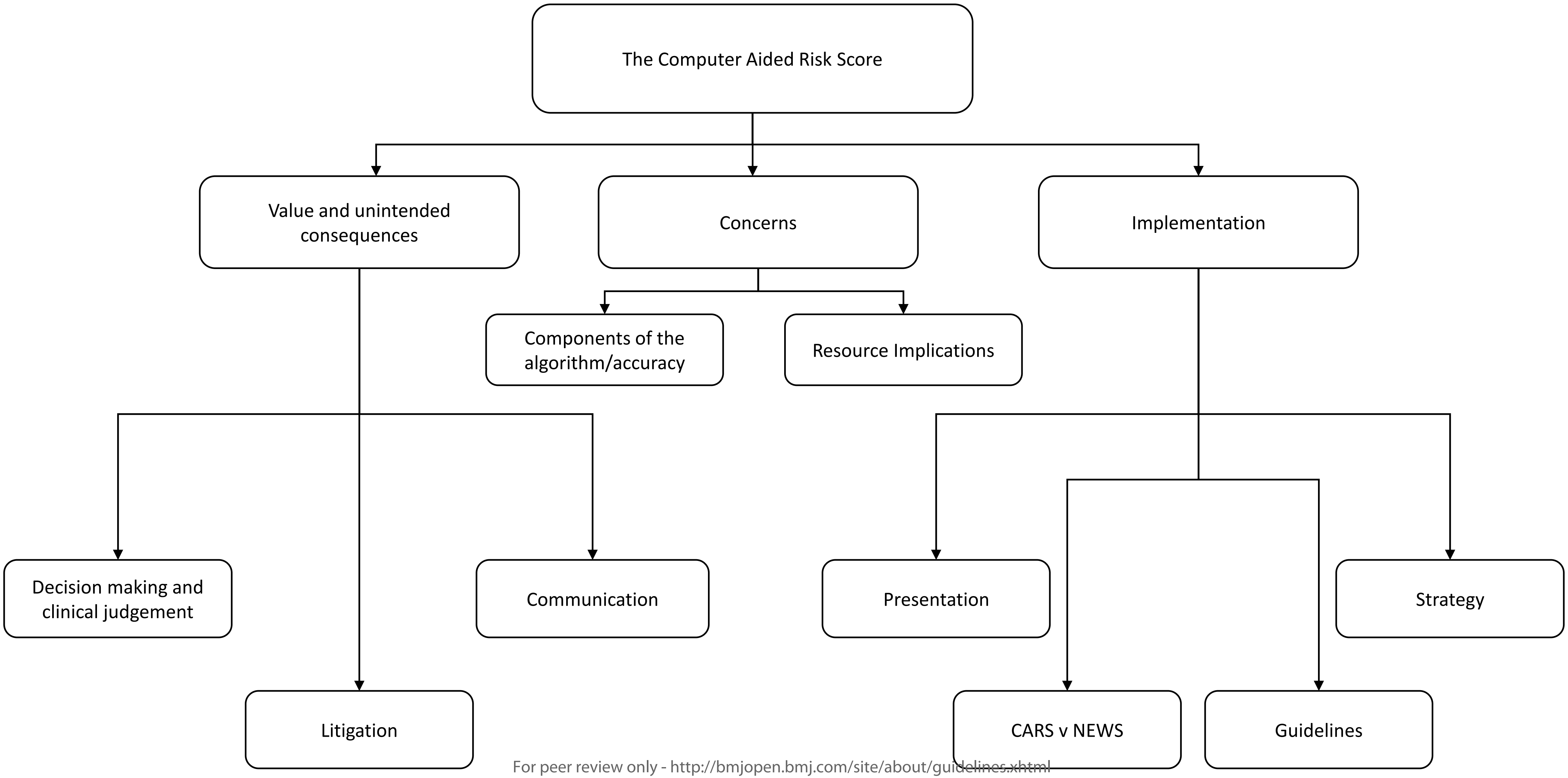

For peer review only - http://bmjopen.bmj.com/site/about/guidelines.xhtml

Standards for Reporting Qualitative Research (SRQR)*

http://www.equator-network.org/reporting-guidelines/srqr/

Title and abstract

	Page/line no(s).
**Title** - Concise description of the nature and topic of the study Identifying the study as qualitative or indicating the approach (e.g., ethnography, grounded theory) or data collection methods (e.g., interview, focus group) is recommended	Page 1 lines 1-3<br>Page 3 lines 1-3
**Abstract** - Summary of key elements of the study using the abstract format of the intended publication; typically includes background, purpose, methods, results, and conclusions	Page 1

Introduction

**Problem formulation** - Description and significance of the problem/phenomenon studied; review of relevant theory and empirical work; problem statement	Page 3
**Purpose or research question** - Purpose of the study and specific objectives or questions	Page 3 para 2 and 3

Methods

**Qualitative approach and research paradigm** - Qualitative approach (e.g., ethnography, grounded theory, case study, phenomenology, narrative research) and guiding theory if appropriate; identifying the research paradigm (e.g., postpositivist, constructivist/ interpretivist) is also recommended; rationale**	Page 4 para 5 and page 5 para 1
**Researcher characteristics and reflexivity** - Researchers' characteristics that may influence the research, including personal attributes, qualifications/experience, relationship with participants, assumptions, and/or presuppositions; potential or actual interaction between researchers' characteristics and the research questions, approach, methods, results, and/or transferability	Last para of page 4 and first para of page 5
**Context** - Setting/site and salient contextual factors; rationale**	Page 4 para's 3 and 4 under the heading "participants"
**Sampling strategy** - How and why research participants, documents, or events were selected; criteria for deciding when no further sampling was necessary (e.g., sampling saturation); rationale**	Page 5 paras 3 and 4 under the title "procedure"
**Ethical issues pertaining to human subjects** - Documentation of approval by an appropriate ethics review board and participant consent, or explanation for lack thereof; other confidentiality and data security issues	Page 4 para 1 under the title "ethical approval"
**Data collection methods** - Types of data collected; details of data collection procedures including (as appropriate) start and stop dates of data collection and analysis, iterative process, triangulation of sources/methods, and modification of procedures in response to evolving study findings; rationale**	Page 5 and 6 under the title "analysis"

For peer review only - http://bmjopen.bmj.com/site/about/guidelines.xhtml

**Data collection instruments and technologies** - Description of instruments (e.g., interview guides, questionnaires) and devices (e.g., audio recorders) used for data collection; if/how the instrument(s) changed over the course of the study	Figures 1 and 2 and page 5 and 6 under the title "analysis"
**Units of study** - Number and relevant characteristics of participants, documents, or events included in the study; level of participation (could be reported in results)	Page 6 under the title "characteristics of the sample" and figure 3
**Data processing** - Methods for processing data prior to and during analysis, including transcription, data entry, data management and security, verification of data integrity, data coding, and anonymization/de-identification of excerpts	Page 5 and 6 under the title "analysis"
**Data analysis** - Process by which inferences, themes, etc., were identified and developed, including the researchers involved in data analysis; usually references a specific paradigm or approach; rationale**	Page 5 and 6 under the title "analysis"
**Techniques to enhance trustworthiness** - Techniques to enhance trustworthiness and credibility of data analysis (e.g., member checking, audit trail, triangulation); rationale**	

Results/findings

**Synthesis and interpretation** - Main findings (e.g., interpretations, inferences, and themes); might include development of a theory or model, or integration with prior research or theory	Page 6 lines 1-2
**Links to empirical data** - Evidence (e.g., quotes, field notes, text excerpts, photographs) to substantiate analytic findings	Page 7 to 12

Discussion

**Integration with prior work, implications, transferability, and contribution(s) to the field** - Short summary of main findings; explanation of how findings and conclusions connect to, support, elaborate on, or challenge conclusions of earlier scholarship; discussion of scope of application/generalizability; identification of unique contribution(s) to scholarship in a discipline or field	Page 12 to 14
**Limitations** - Trustworthiness and limitations of findings	

Other

**Conflicts of interest** - Potential sources of influence or perceived influence on study conduct and conclusions; how these were managed	Page 13 para 3 and page 14 para 1
**Funding** - Sources of funding and other support; role of funders in data collection, interpretation, and reporting	Page 15 under the title "acknowledgements"

*The authors created the SRQR by searching the literature to identify guidelines, reporting standards, and critical appraisal criteria for qualitative research; reviewing the reference lists of retrieved sources; and contacting experts to gain feedback. The SRQR aims to improve the transparency of all aspects of qualitative research by providing clear standards for reporting qualitative research.

For peer review only
For peer review only - http://bmjopen.bmj.com/site/about/guidelines.xhtml

**The rationale should briefly discuss the justification for choosing that theory, approach, method, or technique rather than other options available, the assumptions and limitations implicit in those choices, and how those choices influence study conclusions and transferability. As appropriate, the rationale for several items might be discussed together.

Reference:

O'Brien BC, Harris IB, Beckman TJ, Reed DA, Cook DA. **Standards for reporting qualitative research: a synthesis of recommendations.** *Academic Medicine*, Vol. 89, No. 9 / Sept 2014 DOI: 10.1097/ACM.0000000000000388

For peer review only

For peer review only - http://bmjopen.bmj.com/site/about/guidelines.xhtml
